# Functional surface engineering for cultural heritage protection: the role of superhydrophobic and superoleophobic coatings – a comprehensive review

**DOI:** 10.3762/bjnano.17.6

**Published:** 2026-01-07

**Authors:** Giuseppe Cesare Lama, Marino Lavorgna, Letizia Verdolotti, Federica Recupido, Giovanna Giuliana Buonocore, Bharat Bhushan

**Affiliations:** 1 Institute for Polymers, Composite and Biomaterials, National Council of Research (IPCB-CNR), P.le E. Fermi 1, 80055 Portici, Italy; 2 Dept of Mechanical and Aerospace Engineering, The Ohio State University, 201 W 19th Ave, Columbus, Ohio 43210, USA

**Keywords:** cultural heritage preservation, environmental degradation, functional protective materials, nanocomposite, substrate vulnerability, superhydrophobic coatings, sustainability

## Abstract

The preservation of cultural heritage sites and objects faces critical challenges due to natural aging, environmental degradation, and human-induced damage such as vandalism and graffiti. This review article explores recent advancements in protective strategies for heritage materials including stone, concrete, ceramic, glass, metal, wood, and textiles. Special attention is given to the development and application of superhydrophobic and superoleophobic coatings, which offer promising defense against moisture, pollutants, and oily substances. These functional surfaces, often based on coatings consisting of polymeric, ceramic, and composite materials, can provide durable, non-invasive protection tailored to specific substrate weaknesses and exposure environments (indoor and outdoor). Objective of this review article is to critically examine the most recent studies and materials innovations relevant to cultural heritage site preservation. First the assessment of substrate vulnerabilities and environmental threats is presented, followed by a detailed analysis of coating types and compositions. It concludes with emerging trends, challenges, and future perspectives, offering a valuable resource for researchers, conservators, and materials scientists committed to the long-term safeguarding of historical artifacts and monuments.

## Introduction

**Figure 1:**
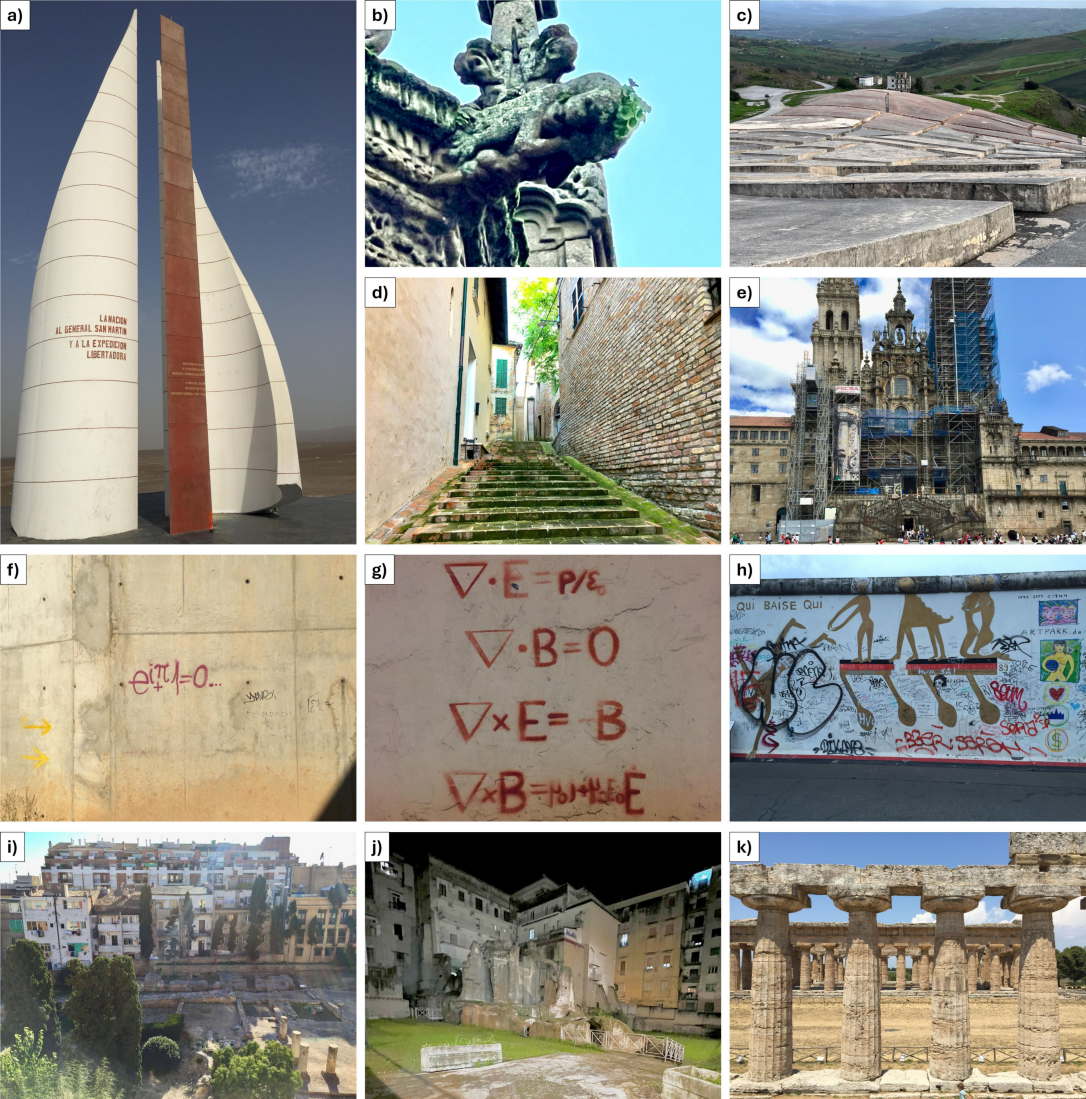
Examples of damage and vandalism occurred on different heritage sites, made of different materials. (a) Corrosion phenomena (most probably due to sea aerosol and bird excreta exposition) of the monument for the General San Martin in Paracas (Perù, 2016); different effects of biofouling occurring on several monuments: (b) detail of the cathedral of Braga (Portugal, 2023), (c) “Cretto di Gibellina” in Sicily region (Italy, 2024), (d) particular of Corinaldo (Ancona, Italy, 2017) historical center, (e) external façade of the cathedral of Santiago de Compostela (Spain, 2017). Examples of graffiti on (f) bridge abutments (2018), (g) buildings (2017) and (h) iconic sites like the East Side Gallery in Berlin (Germany, 2015). Cases of archeological sites exposed to human and environmental actions: (i, j), examples of the interweaving of the modern city with the Roman archeological sites in (i) Baia (2016) and (j) Naples (2021), respectively, both in the Campania region (Italy), and severely exposed to the urban anthropic influence, and (k) the ancient Greek ruins of the open archeological area located in Paestum (Campania, Italy, 2017), severely endangered by environmental influences. All pictures were acquired by the authors during the last decade.

**Figure 2:**
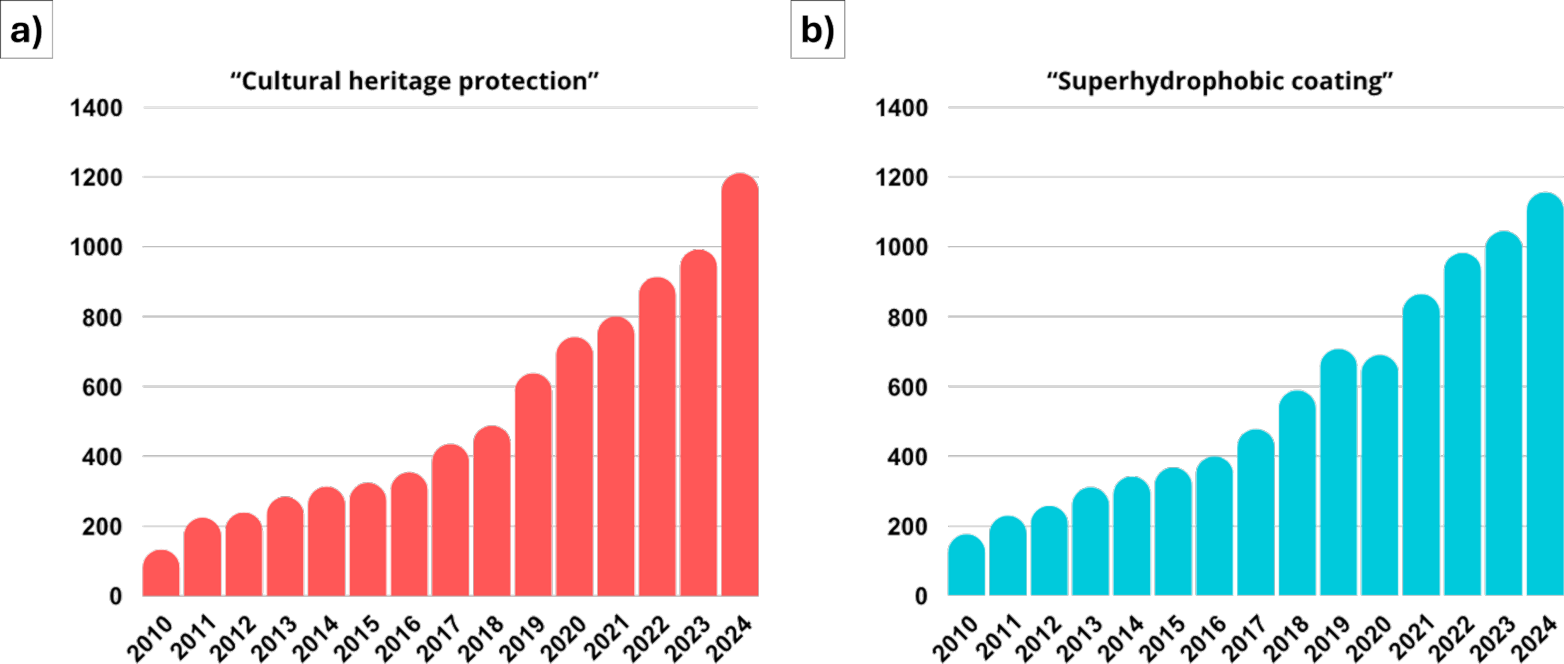
General progression of the total number of scientific papers, books, reviews, and conference papers during the years 2010 to 2024 on (a) “cultural heritage protection” and (b) “superhydrophobic coating” [source: scopus.com, August 2025].

The conservation of cultural heritage is not only an endeavor to preserve the physical integrity of artifacts but also to maintain the cultural identity of societies [1]. With the growing threats posed by environmental factors [2–3] and by human-induced damages, such as mishandling [4] or acts of vandalism [5] (Figure 1), the need for advanced protective coatings has never been more pressing. In Figure 2, the number of publications related to both the cultural heritage preservation (Figure 2a), and to superhydrophobic coatings (from 2010 to 2024, Figure 2b), both showing an outstanding increase over time for both topics.

In 1964, the modern principles of conservation of cultural heritage were conceived on the occasion of the “2nd International Congress of Architects and Technicians of Historic Monuments, Venice, 1964”, as part of the International Charter for the Conservation and Restoration of Monuments and Sites [6], with following statements: “Imbued with a message from the past, the historic monuments of generations of people remain to the present day as living witnesses of their age-old traditions. People are becoming more and more conscious of the unity of human values and regard ancient monuments as a common heritage. The common responsibility to safeguard them for future generations is recognized. It is our duty to hand them on in the full richness of their authenticity”. With the cultural heritage preservation principles written in this way, each person carries a strong collective responsibility to preserve the authenticity of historical testimony. As proof of this heightened perception, since then, more than 9000 scientific papers, review papers, and books have been published in the field of “cultural heritage protection”, 99% of which have been published in the last 30 years [source: scopus.com].

These publications focus on the actions to assess the extent of damage before protecting the artwork. For assessment, several methods can be used, most of which are non-destructive [7]. The papers discussing monitoring and the evaluation of the damages to monuments or landscapes, by means of topographic surveys and successive numerical simulations, are the most cited in this area [8–12]. Probably, one of the most detailed examples is represented by the analysis of the statue of Darius from Susa, in modern Iran [13]. The statue was found in Egypt in 1972, and was then transferred to Teheran, Iran. The paper by Shahrokh reveals how a combination of natural wear, such as erosion and structural stress, and anthropogenic damage, including the loss of the head and deliberate surface abrasion, caused the partial loss of this piece of art. Discovered in a well, its context suggests intentional removal in ancient times, possibly linked to regime change or iconoclastic acts. These findings support the view that the statue’s destruction was not accidental but rather a targeted response to shifting political or cultural dynamics. The study underscores the importance of understanding both environmental and human threats in the preservation of ancient artworks, which also passes through their conservation, when stored or transported, in order to avoid damage in these delicate phases [4].

Just like the analyses of the damaged pieces of art, great attention is devoted to the interventions for their restoration [14], most of which consist of gels and hydrogels [15]. Passaretti et al. [16] analyzed the efficacy of a specifically designed sustainable organogel, composed of poly-3-hydroxybutyrate (PHB), ethyl lactate (EL), and deferoxamine B (DFO), for the simultaneous removal of iron corrosion and aged acrylic coatings from historical metal artefacts. This gel is made of bio-based, biodegradable, and low-toxicity materials, aligned with Green Chemistry principles. Ethyl lactate acts as the organic solvent, PHB as the gelator, and DFO as a chelating agent for Fe(III), all functioning effectively at a mildly acidic pH (~4.8). The authors prepared several mock-ups of mild steel by chemically inducing corrosion and coating with Paraloid^®^ B72, replicating real conservation challenges. Application of the PHB–EL–DFO gel on these mock-ups demonstrated its ability to safely and efficiently remove both corrosion products and polymer coatings, even when coatings penetrated the corrosion layer. The gel was prepared at 110 °C and cooled to form a malleable, easy-to-use material, which could be cut and applied with precision. A control test using a PHB–EL gel (without DFO) confirmed no harmful interaction with the bare steel substrate. Cleaning performance was evaluated for 10, 20, and 30 min of application, and effectiveness was found to increase. Multimodal characterization, particularly micro-FTIR coupled with principal component analysis (PCAn), confirmed the efficiency of the gel and spatially controlled cleaning action. The authors believed that this approach reduces the need for separate treatments and harsh chemicals traditionally used in metal conservation, such as ethylenediaminetetraacetic acid (EDTA) or mechanical abrasion, offering a promising, green innovation for conserving indoor historical iron objects by a targeted removal of both corrosion and old coatings [16]. Although the identification of the impairment and its restoration play crucial roles, the actual focus of the modern conservators and researchers in the field is on the design and the development of innovative materials and techniques to guarantee the safety and the preservation of the selected artwork, preventing possible damages and considering all the potential threats [17].

One of the first testimonies of protection of artworks, in particular of those made of metals, can be traced back to around 50 C.E., in the Naturalis Historia by Pliny the Elder [18], using organic substances (bitumen, pitch, and oil) for preservation purposes. Pliny cited De Agri Cultura, by Cato, acknowledging the conservative merits of amurca, obtained from wastes of olive oil production, that keeps bronze surfaces shinier and free from rust. Moreover, Pliny himself endorsed bitumen for being highly useful for bronze protection against fire, and mechanical and corrosion threats. Other examples of the early evolution of the techniques to protect metal-based sculptures are represented by, again, natural-derived materials like waxes (beeswax among all) and mixtures of wax and resins [19]. However, when wooden sculptures or paintings were considered, linseed oil was applied to prevent cracking and protect the surface [20].

In the contemporary era, starting in the second half of the 20th century, the concern about safety of the cultural heritage has become increasingly prominent. An example of modern protective coating is represented by shellac, the only resin derived from animals, more precisely, from lac insects [21]; it is mostly used for small objects such as furniture. Other conventional materials adopted to protect artworks, mostly used in the 1980s, 1990s, and early 2000s, consisted of other natural resins, like damar resin (derived from damar trees, typical of the southeast Asia [22]), mastic resin (from mastic tree, mediterranean indigenous plant [23]), and copal varnish (derived from tropical trees, mostly East Africa [24]). Each of these resins and varnishes have different characteristics such as high gloss and protection as well as durability and hardness. In the same period, for stone sculptures and building protection, silicone-based consolidants and acrylic resins were adopted [25], offering resistance by penetrating in the porous structure of the artwork surface. As an alternative, together with the protection of stone structures, the path of restoration was also considered, by using lime-based mortars due to their compatibility with historical materials [26]. In those years, reference books were published for conservators, offering detailed descriptions of materials and methods used in the protection and preservation of paintings [27], and, in general, of the chemistry behind the materials for conservation [28]. A still-evolving volume of knowledge is made by The Getty Conservation Institute [29]. In those publications, the principal techniques to be employed in artwork protection and conservation are gathered, with special focus on acrylic-based varnishes and resins, among which Paraloid B-72 has been one of the most extensively used, and is still widely utilized nowadays, even though it easily experiences several ageing effects, such as yellowing due to photo-oxidation processes [30].

In general, protective coatings applied to cultural heritage artifacts must serve as a critical line of defense against environmental degradation and/or human threat (such as vandalism or graffiti). In the last few decades, many preservation materials with specific properties (the most extensively studied are self-cleaning, anti-corrosion, anti-icing, anti-graffiti, anti-fogging, and above all hydrophobicity coatings) have been considered. Some of them showed the feature of being bio-inspired [31–33], and some others have been obtained starting from renewable raw materials [34–35], following the sustainability paradigm [36–39].

The aim of this review article is to summarize relevant papers and materials advancements related to their application in the growing field of cultural heritage protection, with a major focus on superhydrophobic and superoleophobic characteristics. It starts with the substrate materials (stone, wood, metal, ceramics/glass, and textile) and their relative weaknesses and then discusses the threats, which depend on the environment in which the artwork is located (outdoor or indoor). This review article also aims to provide scholars and conservators with a comprehensive overview of current solutions, serving as a foundation for exploring new and innovative approaches to preserving both movable and immovable historical heritage artworks.

## Review

### Coating materials and deposition

The body of scientific literature and technological innovations concerning protective coatings (particularly those exhibiting superhydrophobic properties) has expanded significantly in recent years, especially in high-tech sectors such as electronics, telecommunications, medical devices, automotive, aerospace, and defense [40–42]. This growing interest reflects the critical need to address specific surface-level threats, including icing, fogging, contamination, biofouling, and corrosion [43–44], by leveraging the multifunctional shielding capabilities provided by such advanced coatings. Several review papers provide summary of all these materials and techniques, focusing on applications including aerospace, medical, and automotive. Only in the last years, the importance of cultural heritage applications has been recognized [45], and material science advancements are being applied in the field of prevention, instead of restoration, of the cultural heritage.

At the 15th General Assembly of International Council of Museums (ICOM), held in Buenos Aires (Argentina) in 1986, the “ICOM Code of Professional Ethics”, was unanimously adopted. It has been amended, revised, and retitled as “ICOM Code of Ethics for Museum*s*” in the subsequent years and sets minimum standards of professional practice and performance for museums and their staff [46]. In addition to this document, the “Terminology to characterize the conservation of tangible cultural heritage” resolution was adopted by the international Committee for Conservation of ICOM (ICOM-CC) members during the 15th Triennial Conference held in New Delhi in 2008 since a clear and consistent terminology became necessary [47].

In accordance with these two documents, preventive conservation measures are defined as actions aimed at avoiding or minimizing future deterioration or loss without interfering with the materials or structure of cultural heritage objects. Within this framework, protective coatings employed for the preventive conservation of artworks must fulfil a set of essential requirements to ensure both ethical compliance and technical performance.

First, the coating must be optically transparent and non-intrusive, ensuring that it does not alter visual appearance, color, gloss, or texture of the original surface. Second, it should be reversible or, at minimum, removable without damage, allowing future conservators to return the object to its prior state in line with evolving conservation practices. Third, the coating must be chemically stable and durable under expected environmental conditions, offering long-term protection against light, humidity, pollutants, and other agents of deterioration. Finally, the coating should be safe and ethically acceptable, avoiding the use of hazardous substances and respecting the cultural, historical, and aesthetic significance of the object [46–47]. Over the years, to these established criteria, another aspect has been increasingly, though not yet formally, acknowledged within the conservation community, namely, the sustainability of the material. Along these lines, with broader environmental awareness, coatings are now often expected to minimize ecological impact, both in their production and in their eventual disposal.

In light of these requirements, a wide range of recently developed coatings, including biopolymer-based, nano-engineered, and mineral-inspired solutions, have shown promising protective methods for artworks, offering both ecological, aesthetic, and chemical compatibility. Moreover, since these new preservation solutions are emerging in a historical period marked by heightened environmental awareness (e.g., climate change, pollution, and resource depletion), researchers are striving to develop methods that balance conservation needs with environmental responsibility. Some are the materials substrate to be protected, and some are the solutions adopted up to now [48]. Inspired by the review by Artesani et al., selected scientific papers in the field of cultural heritage protection of the last five years and some other solid background papers of the past decades are herein reviewed.

Several materials are used as coatings for the various types of substrates requiring protection and solve specific issues for each class. As already mentioned, resins (like Paraloid® B72 acrylic resin (B72), Paraloid® B44 acrylic resin (B44) or INCRALAC) and waxes (like Carnauba wax) are still widely used, often in combination with each other, being easily commercially available and well-established solutions for quick metal, glass, or stone artwork protection (especially for frescoes). They have good initial adhesion and transparency; however, several limitations can be highlighted. The resins are not always compatible with metal patina [49] and are also easily prone to yellowing due to ageing from ultraviolet (UV) radiation or thermal stress [50], while the waxes guarantee a protection from corrosion, but only in the early stages of application [51]. For these reasons trend is to use other engineered materials.

The tailoring of coating materials must be guided by the nature of the substrate since porosity, roughness, and coating thickness directly affect adhesion, durability, hydrophobicity, and ultimately compliance with ICOM preventive conservation criteria. Substrate characteristics determine not only how the coating adheres, but also how easily it can be removed, how stable it remains under environmental stress, and whether it alters the optical appearance of the surface. These considerations are particularly relevant when considering different threats. Water is the most pervasive menace, in both indoor and outdoor environments [52]. For stone and other inorganic materials, high porosity and surface irregularities can increase coating absorption, resulting in thicker and less uniform layers that compromise transparency and reversibility, while smoother substrates favor thinner, more controllable films. Furthermore, artworks having stone or inorganic surfaces, may experience erosion due to the effect of acid rain if in outdoor environment (limestone might shift to gypsum due to sulfur dioxide, becoming porous and water-absorbent) [53], while, if in indoor environment, they may experience biological fouling (fungi or bacteria) [54–55], which might initiate bio-deterioration [56]. If the substrate is metallic, corrosion caused by water (rain when outdoor and humidity fluctuation when indoor) is the main issue, causing rusty surfaces due to oxygen dispersed in water or to salt residues [57]. For these reasons, much effort is devoted to developing coatings that minimize water interaction, while ensuring that substrate-dependent properties such as porosity, roughness, and coating thickness are carefully considered, as these decisively govern the effectiveness and compatibility of any protective layer.

For completeness, there are several cooperating factors that cause damage: (i) the environment, outdoor or indoor; (ii) single threats such as water, temperature, or air pollution; (iii) the intrinsic vulnerability of the material through porosity and microstructure; and (iv) the chemical composition of the material itself, that is, stone, wood, metal, or ceramic. For this reason, it is necessary to fix a single factor and evaluate the variation of the others. Herein, we have chosen the substrate material to be fixed, evaluating how each solution has been adopted to prevent each risk, in a case-by-case subdivision.

### Mineral-based materials

**Stone substrates.** Stone substrates are among the most extensively studied surfaces in the field of cultural heritage protection (for example, historical constructions and buildings, as well as marble statues both in outdoor and indoor environments). Many approaches are used to solve several issues arising from their intrinsic properties such as porosity, which may cause fungal or bacterial fouling [58], and surface chemistry and the affinity to water and humidity [59]; also, there are varnishes against graffiti vandalism [60]. In Table 1, different types of heritage mineral-based substrates, along with the base materials of the successfully applied coatings, the resulting functional properties, and the respective ICOM criteria (transparency “Tr”, stability “St”, safety “Sa”, reversibility “Re”), are reported.

**Table 1:** Coating materials used for heritage mineral-based substrates (ceramic, stone and glass), with their functional properties.

Application substrate(s)	Coating materials	Feature	ICOM criteria (+1)^a^					Ref.
			Tr	St	Sa	Re	Su	

Lecce stone (calcite-rich porous stone)	water-based fluoropolymer	eco-friendly, water-repellent, graffiti-resistant, vapor-permeable	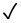	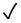	~	×	~	[60]
marble (Thassos and Penteli), potentially limestone	hydroxyapatite (HAp) nanocrystals + polyelectrolyte multilayers	hydrophilic, self-healing, tunable adhesion, weathering resistance	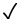	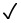	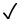	~	~	[61]
Lecce stone	fluoropolymer modified with SiO_2_; commercial fluorine/silicon-based polymers	bird excreta resistance, oleo/hydrophobicity, anti-graffiti, high durability	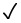	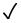	~	×	~	[62]
granite, marble, limestone	chitosan + microcrystalline cellulose (MCC) + oregano essential oil (OEO)	antimicrobial, biodegradable, low solubility/swelling/wettability	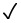	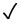	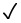	~	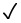	[63]
concrete (cement-based substrates)	PDMS^b^ + vacuum ceramic microbeads (VCMs) modified with poly(catecholamine)	thermal insulation, hydrophobicity, abrasion/chloride resistance	×	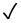	~	×	~	[64]
mortar, cementitious materials	TiO_2_ + carbon dots in TEOS^c^-PDMS silica matrix	self-cleaning, photocatalytic, UV–visible light activity	~	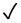	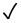	×	~	[65]
ceramic (Cucuteni pottery)	AMF coating (TMSPMA^d^ + TFPTMS^e^-derived nanostructured silsesquioxane) vs Paraloid^®^ B72	UV protection, low degradation, color stability	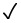	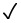	~	#	~	[66]
geopolymers (ceramic-like substrates)	sol–gel alkoxysilanes (varied chain length and halogens)	hydrophobicity, mechanical strength, heat resistance	#	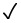	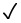	×	~	[67]
Portuguese glazed tiles	TiO_2_ sol–gel coating	anti-biofouling (ineffective), chemical interaction with substrate	×	×	~	×	~	[68]
terracotta (outdoor sculptures)	fluorinated copolymer (PVDF-HFP^f^), alkylpolysiloxane (PSw), n-SiOR^g^, wnf-SiO_2_^h^	water repellency, durability, surface stability, aesthetic preservation	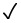	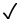	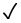	~	~	[69]

^a^Tr: optical transparency and non-intrusiveness (the coating should not alter appearance, color, gloss, or texture); St: chemical stability and durability (the coating is resistant to environmental agents like light, humidity, and pollutants); Sa: safety and ethical acceptability (no hazardous substances during coating application, and respect for cultural/historical significance); Re: reversibility or removability (the coating can be removed without damage, allowing future conservators to restore the object to its prior state); Su: sustainability (preference for environmentally responsible materials and processes, reducing the ecological footprint throughout the coating’s life cycle). 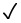: yes; ×: no; ~: partially; #: unclear; ^b^PDMS: polydimethylsiloxane; ^c^TEOS: tetraethoxysilane; ^d^TMSPMA: 3-(trimethoxysilyl)propyl methacrylate); ^e^TFPTMS: (3,3,3-trifluoropropyl)trimethoxysilane; ^f^PVDF-HFP: poly(vinylidene fluoride-*co*-hexafluoropropylene; ^g^n-SiOR: dispersion of functionalized silica nanoparticles; ^h^wnf-SiO_2_: nanostructured silica gel in hydroalcoholic solution.

Regarding stone artworks, there have been many examples in previous years in which the surfaces have been repaired using inorganic materials, either compatible to or even mimicking the original material [70], with some attempts in the present decade [71]. For this reason, examples of investigation of inorganic coatings are quite limited. However, Hafez et al. [61] explored nanostructured hydrophilic and self-healing hybrid coatings based on hydroxyapatite (HAp) nanocrystals and polyelectrolyte multilayers, formed in situ on Greek marble (Thassos and Penteli) using a simple layer-by-layer spray surface functionalization technique. The authors claimed that the components were water-soluble, easily accessible, and with the possibility to tune properties like adhesion, self-healing, and weathering resistance by optimizing the procedure for their fabrication. Thus, using the ICOM nomenclature, and accordingly to the reported results, the hydroxyapatite–polyelectrolyte multilayer system fulfils several key conservation criteria. It provides optical transparency with minimal alteration of the substrate’s appearance, demonstrates high chemical stability and durability under acid exposure, weathering, and salt crystallization, and is composed of nontoxic, biocompatible constituents, thus addressing safety and ethical considerations. Reversibility is only partially achieved as removal is possible under certain conditions but cannot be guaranteed without potential residues. Sustainability is also partially met; the process is water-based and free from hazardous reagents, yet its reliance on synthetic polyelectrolytes reduces its ecological advantage compared to fully bio-derived alternatives.

**Figure 3:**
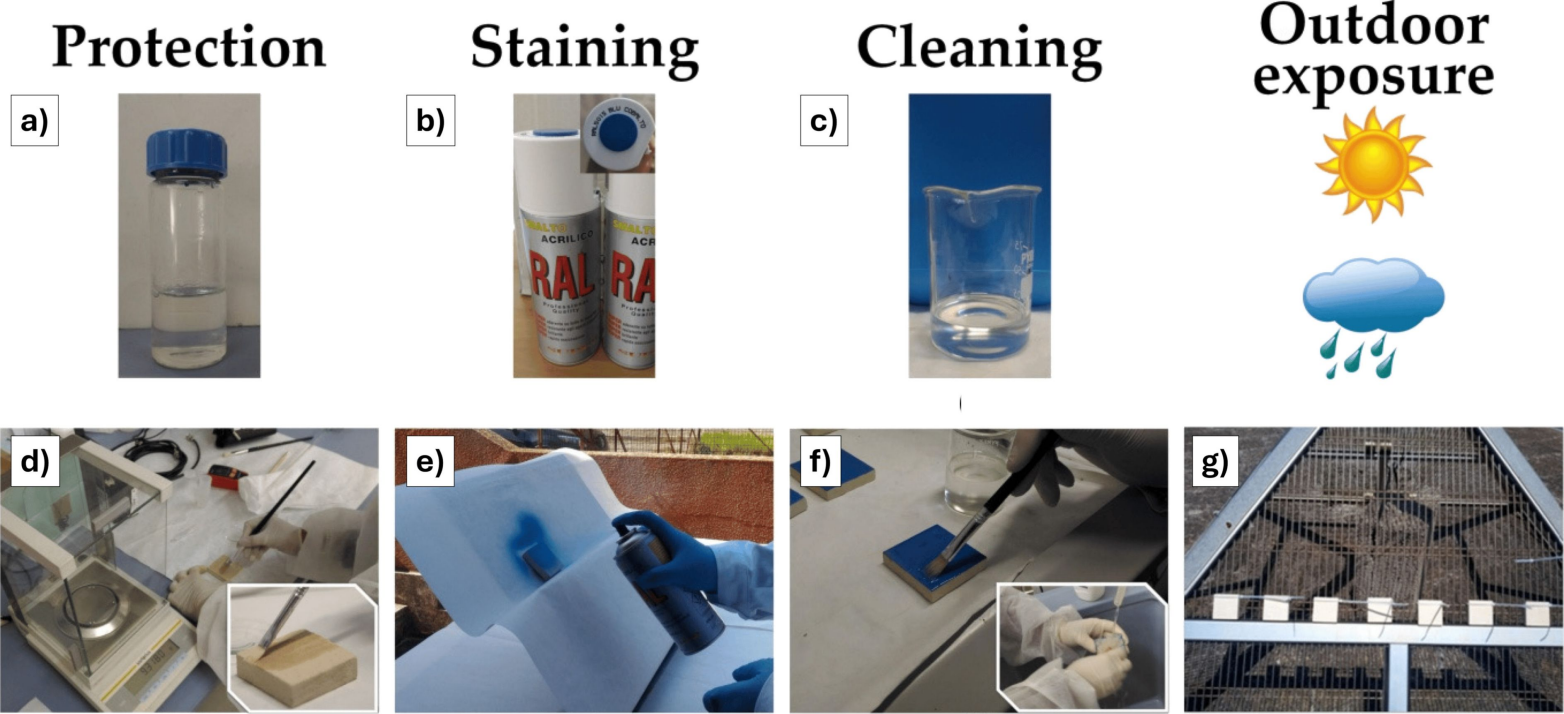
Materials and treatments. (a) Liquid fluorine-based polymer for stone protection; (b) spray cans of acrylic paint; (c) chemical remover for graffiti cleaning; (d) setup for the application of the coating and detail of brushing on a specimen of Lecce stone; (e) staining with blue paint; (f) application of the chemical remover and subsequent rinsing with running tap water and a sponge; (g) stone specimens during outdoor exposure. Figure 3 was reproduced from [60] (© 2021 M. Lettieri et al., published by MDPI, distributed under the terms of the Creative Commons Attribution 4.0 International License, https://creativecommons.org/licenses/by/4.0).

There are reported examples of polymer-based coatings. Concerning the outdoor environment for preservation of inorganic materials used in cultural heritage monuments and artworks from damages, such as alveolation, erosion, rising damp, or salt crystallization, Lettieri et al. [60] presented a partially eco-friendly, water-based fluoropolymer coating to protect porous stones like Lecce stone, commonly used in cultural heritage structures (mainly constituted by calcite). This coating effectively reduces water absorption and resists graffiti staining, helping to preserve the material from environmental and human-induced damage. Importantly, it does so without altering the stone’s appearance as confirmed by minimal color changes in treated samples (Figure 3). The coating also maintains the stone’s vapor permeability, which is essential for its long-term durability. Short-term outdoor exposure tests showed that the protective effects remain stable, making it a promising solution for heritage conservation. The same group also investigated a way to protect the same surfaces from simulated bird excreta [62], comparing the effectiveness of an experimental fluoropolymer (modified with SiO_2_ particles) with two commercial polymers (a fluorine-based and a silicon-based). The experimental coatings exhibited superior durability, being able to provide oleo/hydrophobic and anti-graffiti properties, also withstanding the action of bird excreta. Regarding the ICOM criteria, both studies demonstrate that the criteria of transparency and stability are satisfactorily achieved, as the investigated polymeric and nanoparticle-filled coatings provide effective protection for stone substrates without significant alteration of their aesthetic properties and with considerable resistance to environmental stressors. However, the criterion of reversibility is not addressed in either work, representing a critical shortcoming in relation to internationally recognized conservation principles. The aspect of safety is only partially considered, generally limited to substrate compatibility or the mention of eco-friendly solvents, without a comprehensive evaluation of potential toxicological or environmental risks. With respect to sustainability, neither study explicitly integrates this parameter as a design or assessment criterion. Although occasional references to environmentally friendly formulations or solvent substitutions are made, a systematic consideration of life-cycle impacts and ecological footprint is absent.

Even if fluorine-based polymers were the best solution for heritage protection, there are concerns, especially for the strong environmental threat they may cause, due to their endurance, their resistance to external factors, and the difficulty of being degraded once polymerized. Silva et al. [63] investigated a single solution to solve such environmental issues by using chitosan coatings filled with essential oils, also to impart a bactericidal effect without the use of biocides or synthetic materials. In addition, they added microcrystalline cellulose (MCC), to the initial mixture in order to reduce the wettability of the final coating. The formulation containing 2% of MCC enhanced with oregano essential oil (OEO) showed the lowest solubility, swelling, and wettability, along with strong antimicrobial activity against stone-deteriorating microorganisms. The tested microorganisms were three bacterial strains, that is, *Staphylococcus aureus*, *Bacillus cereus*, and *Pseudomonas aeruginosa*, one fungus strain (*Penicillium chrysogenum*) and one yeast strain (*Rhodotorula* spp.). Specifically, the microbial activity was reduced by 100% for *Staphylococcus aureus*, *Pseudomonas aeruginosa*, and *Rhodotorula* spp., while only partial inhibition was observed for the two other tested organisms.

When applied to granite, marble, and limestone, the coating formed a protective but uneven layer that reduced surface wettability without altering the stones’ appearance. These results highlight the potential of chitosan-MCC-OEO coatings as biodegradable alternatives for stone conservation with low risk regarding human safety. Moreover, a majority of the ICOM criteria is fulfilled. The chitosan-MCC-OEO formulation is optically transparent and non-intrusive, ensuring no significant alteration of color or gloss. In terms of reversibility, while the material formed a stable film, the study did not provide conclusive evidence regarding its removability without affecting the substrate, which remains a limitation. Stability was demonstrated, with the films retaining integrity under immersion, showing low solubility, and conferring antimicrobial protection. Regarding safety, both chitosan and oregano essential oil are recognized as nontoxic to humans, biocompatible, and safe for handling. Finally, the criterion of sustainability was addressed, as the materials are biodegradable, renewable, and derived from low-impact sources.

**Cement-based substrates.** Cement-based materials are widely used in many buildings and constructions of historical and cultural significance. Wang et al. [64] presented the shielding of a historical building from simulated urban outdoor environment. They presented a multifunctional thermal insulation coating incorporating modified vacuum ceramic microbeads (VCMs) to enhance energy efficiency and protection of concrete structures. The VCMs were modified through the polymerization of catechol and hexamethylene diamine, forming a thin poly(catecholamine) (PCA) layer. Subsequently, the authors incorporated the PCA-coated VCMs into a polydimethylsiloxane (PDMS) matrix, significantly reducing the thermal conductivity of the coatings and improving their surface hydrophobicity. The coating provided robust protection for concrete, enhancing abrasion resistance and interfacial adhesion under various environmental conditions, including immersion in saline solutions. It also effectively reduced chloride ion penetration, contributing to the durability of concrete substrates. Additionally, the coating was also able to promote temperature regulation by lowering surface temperatures, demonstrating its potential for passive cooling and material preservation in architectural heritage.

**Figure 4:**
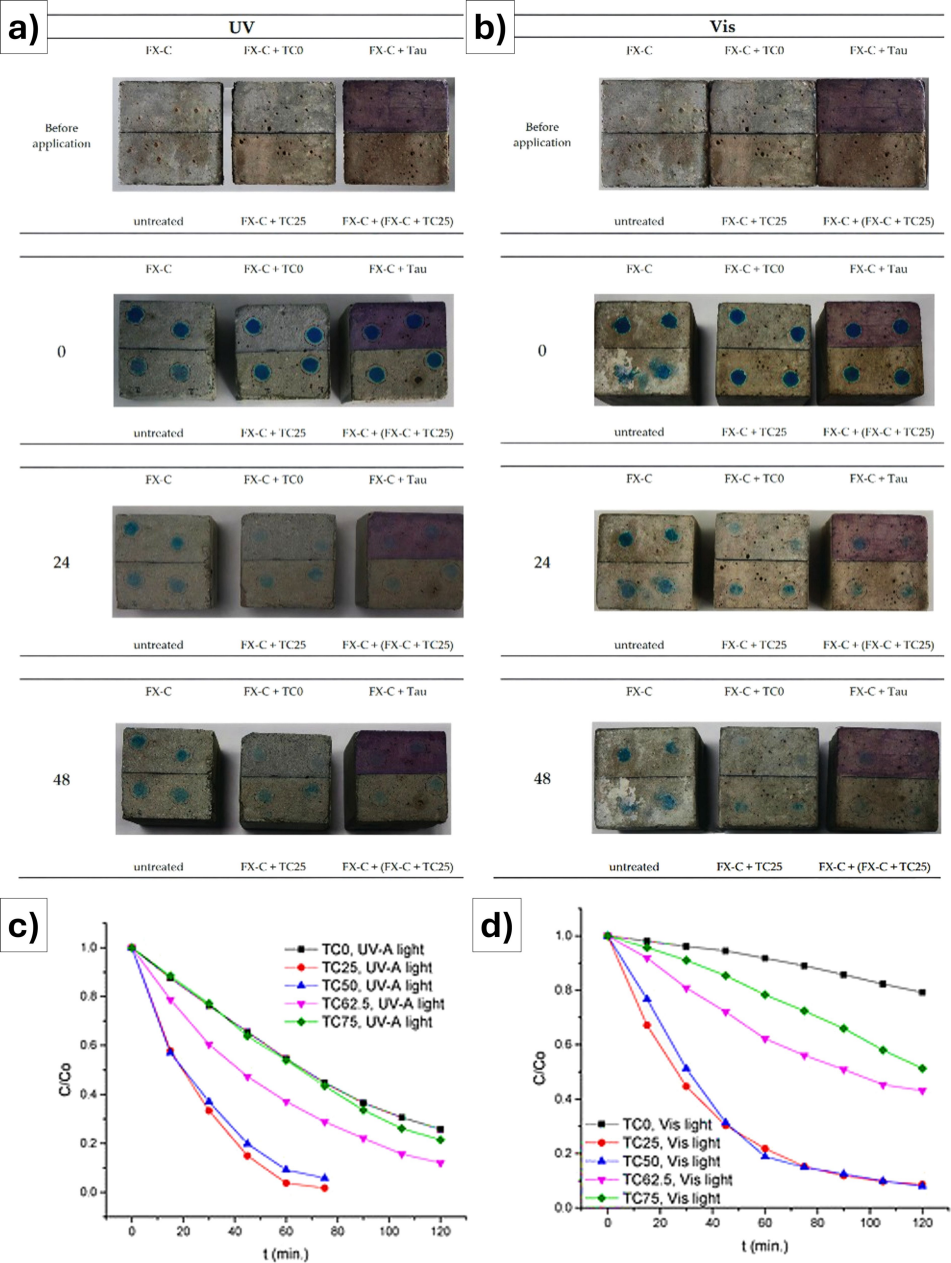
Macroscopic evaluation of the gradual MΒ degradation on treated (by means of an advanced hydrophobic consolidant, designated as FX-C, using a sol–gel process that combines TEOS, PDMS and nanoscale calcium oxalate (nano-CaO) for the preservation of cultural heritage modified with TiO_2_/C-Dots hybrids) and untreated cement mortars, before and after methylene blue (MB) application, under (a) UV-A and (b) visible light irradiation. Photocatalytic self-cleaning performance of TiO_2_–C-dot composites under (c) UV-A and (d) visible light irradiation. Under UV-A exposure, TC25 and TC50 exhibited the highest methylene orange (MO) degradation rates (> 90%) compared to pristine TiO_2_ (TC0) and composites with higher carbon content. A similar trend was observed under visible light, though with slower kinetics. These results indicate that moderate C-dot incorporation enhances photocatalytic efficiency, while excessive loading reduces performance. Figure 4 was reproduced from [65], (© 2022 C. Gryparis et al., published by MDPI, distributed under the terms of the Creative Commons Attribution 4.0 International License, https://creativecommons.org/licenses/by/4.0).

Embedding specific functional fillers into thin layers is the best way to quickly endorse conventional and tailored polymers, possibly already widely used in the field of cultural heritage protection. Gryparis et al. [65] investigated self-cleaning photocatalytic coatings to protect cementitious materials from environmental pollutants (i.e., methylene blue (MB) and methyl orange (MO)). Titanium dioxide, known for its photocatalytic properties, was combined with carbon dots (C-dots) to enhance activity under both UV-A and visible light. The TiO_2_/C-dot composites were synthesized via a green, hydrothermal method using citric acid and hydroxylamine, resulting in anatase-phase particles. Various C-dot loadings were tested; composites with moderate content (i.e., TC25 and TC50) demonstrated superior degradation of MO dye under simulated solar and natural sunlight, while higher C-dot loadings (TC62.5 and TC75) reduced performance, likely due to surface-masking effects. To fully exploit the photocatalytic activity of the TiO_2_/C-dot composites on cementitious substrates, they were embedded in a silica-based consolidant composed of tetraethoxysilane (TEOS) and PDMS, forming a protective and functional coating. The film structure fully integrated the C-dots, without altering TiO_2_ bonding, maintaining visual transparency and color neutrality on mortar surfaces. Under both UV and visible light, the coated mortars exhibited self-cleaning behavior by breaking down organic pollutants (Figure 4a,b). The optimal formulations preserved activity outdoors and under accelerated ageing conditions (Figure 4c,d). Overall, the adopted methodology did not require heavy metals and employed biodegradable, low-cost precursors. These results highlight how tailored C-dot loading and hybrid silica matrices can significantly enhance the photocatalytic protection of cement-based materials.

These two papers show materials with clear strengths in terms of stability since both coatings demonstrate long-term durability under relevant environmental conditions. Human safety is generally respected, with one [65] study employing heavy-metal-free components and the other [64] not reporting major hazards, though without extensive toxicological testing. Sustainability is addressed in both cases, either through energy savings during use or through low-cost and “green” synthetic approaches, but neither provides a full life-cycle perspective. By contrast, the criteria of optical transparency and reversibility are not convincingly met, as both coatings alter surface appearance to some degree and do not offer practical removal strategies.

**Ceramic/glass substrates.** In the broad category of inorganic substrates used in artworks, in addition to stone- and cement-based materials, ceramics and glass objects can also be included. Although pottery objects are continuously unearthed in excavation fields, there are few studies on innovative ways to protect them. Due to their small dimensions, many are stored in the controlled environment of museums and expositions, thus traditional protective materials are often considered sufficient. However, in some cases, when traditional protection is not sufficient (e.g., under UV radiation), innovative steps must be taken. In the study by Oancea et al. [66], Neolithic Cucuteni ceramic pottery was the focus of investigation. Two types of water-repellent coatings were compared, namely, the conventional Paraloid^®^ B72 and a synthesized nanostructured material (AMF). The latter was produced via hydrolysis and polycondensation of 3-(trimethoxysilyl)propyl methacrylate (TMSPMA) and (3,3,3-trifluoropropyl)trimethoxysilane (TFPTMS) in the presence of a dodecylamine surfactant, yielding a crack-free nanostructured film incorporating silsesquioxane, methacrylate, and fluorine units. The comparison focused on UV-induced degradation, assessing the coatings’ efficiency in UV protection. B72 exhibited significant photodegradation, with an increase in carbonyl index, indicating the formation of oxidation products via chain scission and radical reactions, while AMF showed a decrease in carbonyl index, suggesting crosslinking and a higher resistance to degradation due to the formation of ladder-like silsesquioxane structures. Furthermore, it was observed that B72 was more prone to exposing reactive groups to UV light, whereas AMF’s surface structure helped to shield reactive groups. This effect was further supported by its more homogeneous AMF surface after prolonged UV exposure, which also contributed to maintaining a nearly constant, relatively high contact angle.

Rather than organic polymers, inorganic polymers, also known as geopolymers, are often used with this class of artwork. This is due to the fact that geopolymers, principally made of aluminosilicates, are materials chemically similar to ceramics [72]. In their study, Ielo et al. [67] developed and tested hybrid sol–gel coatings based on alkoxysilanes with varying molecular structures. Two different functionalization strategies were used. One involved a surface treatment, where sol–gel coatings were applied post-synthesis to the dry geopolymer monoliths. In the other, a bulk modification, alkoxysilanes were directly added to the geopolymeric mixture before curing. The two routes differed in how the alkoxysilanes were made, especially regarding hydrocarbon chain length and halogen content, which influenced their interaction with the geopolymer matrix. Both materials showed increased hydrophobicity, particularly when using long-chain or halogenated alkoxysilanes, which helped chemical bonding between the hybrid coatings and the geopolymer structure. However, since the bulk-modified samples exhibited a more compact microstructure, mechanical tests resulted in an increased compressive strength for these samples, suggesting stronger structural reinforcement, an improved heat resistance, and a reduction in open pores and surface area, contributing to lower permeability and greater environmental resistance, thanks to the sol–gel-derived procedures.

**Figure 5:**
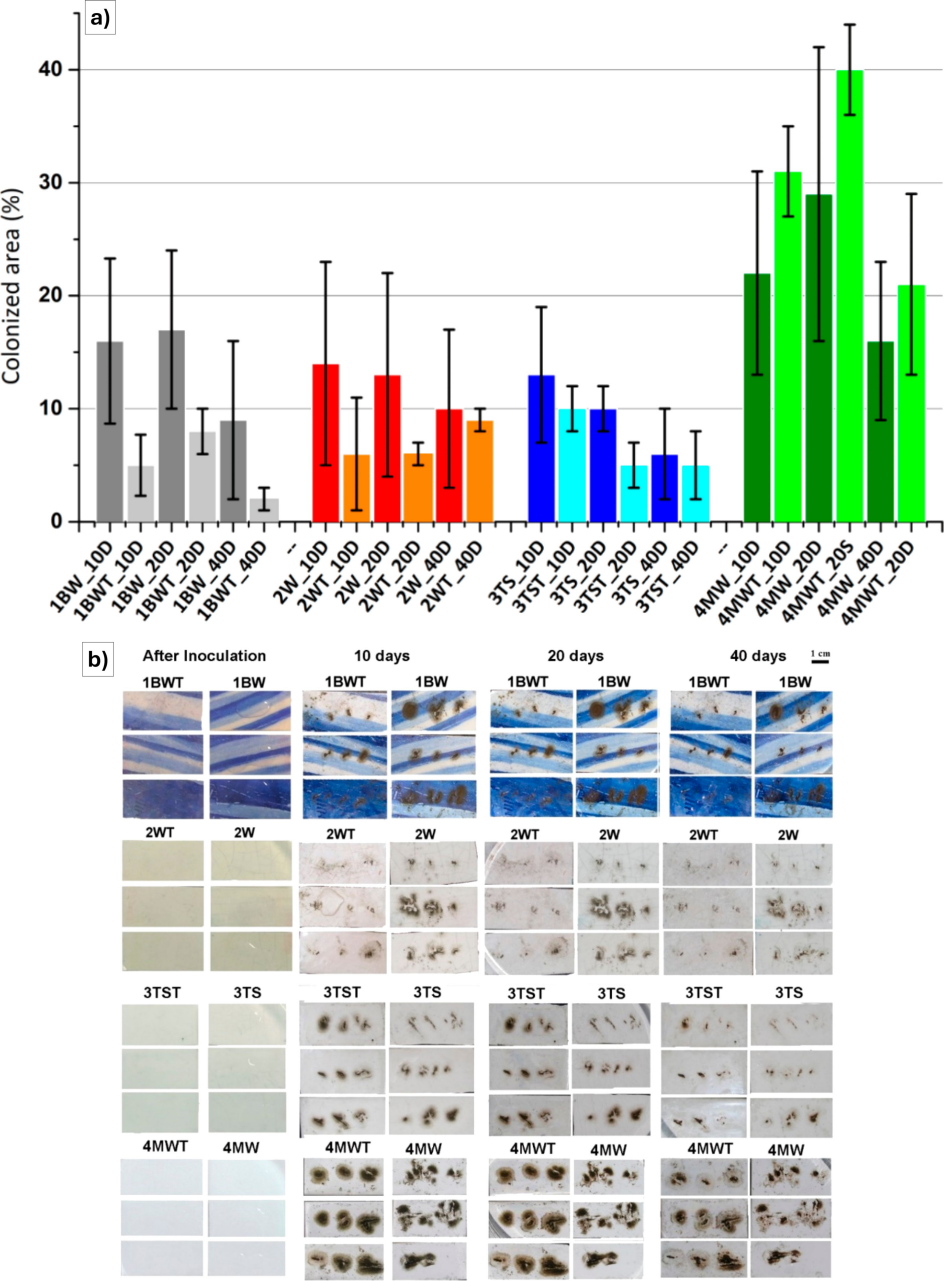
(a) Average percentage and standard deviation of tile surface (%) colonized by the fungus *Cladosporium* sp. on non-treated (*n* = 3, darker color) and TiO_2_-treated (*n* = 3, lighter color) tiles obtained by digital image analysis after 10, 20, and 40 days of incubation. (b) Photographic documentation made during the experiment of fungal growth on glaze tile samples 1BW (non-treated), 1BWT (TiO_2_-treated), 2W (non-treated), 2WT (TiO_2_-treated), 3TS (non-treated), 3TST (TiO_2_-treated), 4MW (non-treated), and 4MWT (TiO_2_-treated) at four different stages, that is, after inoculation, after 10 days of incubation, after 20 days of incubation, and after 40 days of incubation. Scale bar: 1 cm (upper right). Figure 5 was reproduced from [68], (© 2020 M. Coutinho et al., published by MDPI, distributed under the terms of the Creative Commons Attribution 4.0 International License, https://creativecommons.org/licenses/by/4.0).

A sol–gel strategy was also considered in [68], in which TiO_2_ was used in inorganic coating for the prevention of biological attachment, particularly from fungi, on Portuguese glazed tiles (Figure 5). Four different tile substrates were selected for the deposition of sol–gel thin coatings, namely, 1BW, 2W, 3TS, and 4MW. These tiles were chosen to represent a range of manufacturing techniques, including a hand-painted, blue and white earthenware tile (1BW), a semi-industrially produced stencil-decorated tile (2W), an industrially manufactured white earthenware tile (3TS), and a contemporary mass-produced tile (4MW). As a result, the authors claimed that the technique was not suitable for such applications even if the proliferation of the chosen fungus (*Cladosporium* sp.) was slightly reduced upon the application of the coating on the examined tiles (Figure 5a), the aesthetics, in terms of surface colors, was altered, as well as the mineralogical composition, since the TiO_2_ layer chemically interacted with the glazed substrate (Figure 5b). This posed risks to the physical integrity of the artwork and also raised concerns about long-term impacts on historic material.

Among ceramic materials constituting artworks, terracotta suffers most from several threats, especially when in outdoor environment. An example is the bas-relief “Muro del Vento” by Domenico Matteucci, made in 1987 and exposed in the open air museum in Faenza, Italy [69]. Although there are several commercial coatings, only few are targeted for terracotta, which is a porous and highly degradation-prone material. Spadavecchia et al. tested four commercial coatings, originally designed for natural and artificial stones, namely, a fluorinated copolymer (poly(vinylidene fluoride-*co*-hexafluoropropylene, PVDF-HFP), an aqueous emulsion of alkylpolysiloxane (PS_W_), a dispersion of functionalized silica nanoparticles (n-SiOR), and a nanostructured silica gel in hydroalcoholic solution (wnf-SiO_2_). Terracotta specimens chemically and physically matching the original artwork were fabricated and treated with each coating.

The coated and uncoated specimens were exposed to accelerated ageing through acid rain runoff simulation and climatic chamber exposure, alongside a long-term outdoor weathering test. Each coating had pros and cons. The fluorinated copolymer reduced wettability but failed to limit water absorption and showed poor resistance under rain exposure, similar to untreated samples. In contrast, the silicon-based coatings demonstrated better long-term hydrophobicity and surface stability. Among them, PS_W_ slightly enhanced water retention control, while wnf-SiO_2_ offered the most effective and durable protection, with minimal aesthetic alteration. n-SiOR, although initially effective, induced noticeable color change and surface alteration after exposure.

To summarize, bearing the ICOM criteria, there are several observations. The study on Paraloid^®^ B72 and AMF for Cucuteni ceramics [66] demonstrated good transparency and long-term stability, though reversibility is uncertain, safety was only partly considered, and sustainability is limited to performance aspects. Hybrid sol–gel coatings on geopolymeric supports [67] showed stability and chemical resistance within a green-chemistry framework, but transparency and reversibility were not convincingly addressed. The TiO_2_ sol–gel films on Portuguese glazed tiles [68] failed to ensure transparency and stability as they induced aesthetic alterations and did not prevent biodeterioration; reversibility was lacking, and safety and sustainability were only partially met. In contrast, the comparative study on commercial coatings for terracotta [69] showed silica-based formulations as stable, protective, and compatible, with reasonable transparency and partial reversibility, although sustainability remained unexplored. Overall, stability and partial safety are the most consistently fulfilled criteria, while reversibility and sustainability remain weakly developed. Transparency depends strongly on the formulation, with silica-based and acrylic systems performing better than TiO_2_ sol–gels. Sustainability is usually invoked through eco-friendly synthesis or functional performance, yet none of the works provide full life-cycle or disposal assessments, leaving this criterion only partially addressed.

Less attention is devoted to the protection of glass-based substrates. The efforts devoted to the protection of such substrates are mainly focused on providing superhydrophobic properties of the artworks (see the dedicated section on superhydrophobic coatings).

### Metal substrates

Metals are exposed to a wide range of threats from the environment, as well as from human actions. Thus, approaches have been taken by several study groups to solve these issues [73–74]. In Table 2, various types of heritage metal-based substrates, the base materials of the successfully applied coatings, and the resulting functional properties are presented.

**Table 2:** Coating materials used for heritage metal-based substrates along with their functional properties.

Application substrate(s)	Materials/composites	Feature	ICOM criteria (+1)^a^					Ref.
			Tr	St	Sa	Re	Su	

bronze artifacts (outdoor environment)	fluorinated waterborne polyurethane (F-WPU) + ZIF-8^b^ (MOF^c^) + Ti_3_C_2_T*_x_* (MXene^d^) + PFDTES^e^	anticorrosion, hydrophobic (WCA^f^ = 154°), self-cleaning, UV-stable, reversible, high impedance, low corrosion current	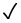	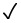	~	~	~	[75]
bronze (outdoor artworks)	modified Paraloid^®^ B44 with AEDTA^g^, MPT^h^, BTA^i^ + light stabilizers	enhanced sustainability, corrosion resistance, inhibitor retention, photo-stability	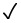	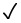	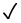	~	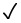	[76]

^a^Tr: optical transparency and non-intrusiveness (the coating should not alter appearance, color, gloss, or texture); St: chemical stability and durability (the coating is resistant to environmental agents like light, humidity, and pollutants); Sa: safety and ethical acceptability (no hazardous substances during coating application, and respect for cultural/historical significance); Re: reversibility or removability (the coating can be removed without damage, allowing future conservators to restore the object to its prior state); Su: sustainability (preference for environmentally responsible materials and processes, reducing the ecological footprint throughout the coating’s life cycle). 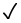: yes; ×: no; ~: partially; #: unclear; ^b^ZIF-8: commercial MOF; ^c^MOF: metal organic framework; ^d^MXene: two-dimensional inorganic compound; ^e^PFDTES: 1*H*,1*H*,2*H*,2*H*-perfluorodecyltriethoxysilane; ^f^WCA: water contact angle; ^g^AEDTA: 5-ethyl-1,3,4-thiadiazol-2-amine; ^h^MPT: 5-mercapto-1-phenyltetrazole; ^i^BTA: benzotriazole.

**Figure 6:**
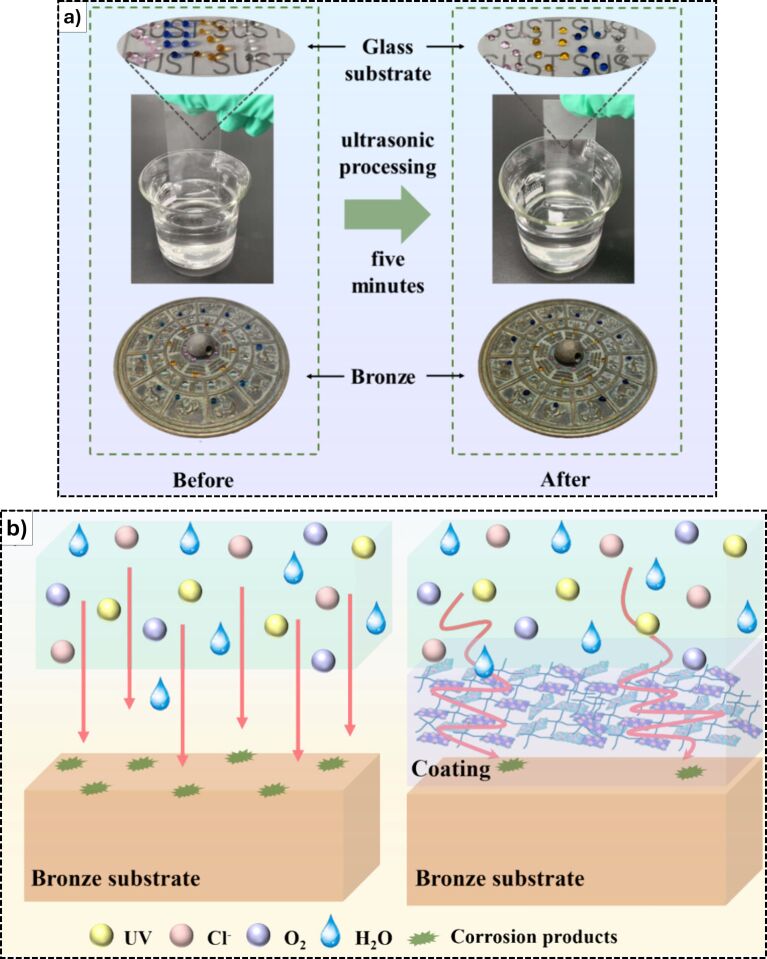
(a) Application of the F-WPU@ZIF-8/Ti_3_C_2_T*_x_* coating for cultural heritage conservation. (b) Corrosion mechanisms on the bronze substrate (left) without and (right) with the F-WPU@ZIF-8/Ti_3_C_2_T*_x_* coating. Figure 6 was adapted with permission from [75], Copyright 2025 American Chemical Society. This content is not subject to CC BY 4.0.

Regarding metal protection, corrosion is the first threat to be considered. Wu et al. [75] focused on those dangers for bronze statues, which may corrode once unearthed, even if the piece of art is covered with anticorrosive coatings. Barriers between corrosive media and bronze may present discontinuities or micropores, through which oxygen, water, or ions (e.g., Cl^−^) can reach the metal surface. In Figure 6a, the production process and final application of a novel organic–inorganic composite coating, designed to provide advanced anticorrosion protection for bronze artifacts is described. The coating combines waterborne polyurethane (WPU) with the nanomaterials ZIF-8 (a metal-organic framework, MOF) and Ti_3_C_2_T*_x_* (a 2D MXene, whose use was considered to enhance the tortuosity of the paths that gases, liquids, and ions must follow to reach the metal substrate [77]). PFDTES was used to introduce fluorinated groups for enhanced hydrophobicity. The final product was labeled F-WPU@ZIF-8/ Ti_3_C_2_T*_x_*. The two nanomaterials (the MOF ZIF-8 and the 2D MXene Ti_3_C_2_T*_x_*), synthesized and dispersed uniformly before being incorporated into the WPU matrix and sprayed onto the bronze substrates, were aimed to fill microcracks and structural defects in the polymer matrix, improving coating density and physical barrier performance (ZIF-8 helps prevent Ti_3_C_2_T*_x_* aggregation and chemically cross-links with WPU, enhancing mechanical integrity). The final cross-linked coating had a water contact angle of about 154°, indicating high water repellency and self-cleaning capabilities. More importantly, electrochemical tests showed an impedance significantly higher than other coatings, and a corrosion current (*i*_corr_) value two orders of magnitude lower than that of control coatings, confirming excellent anticorrosion performance. In addition, in UV aging tests, the coating maintained stability and hydrophobicity after 15 days of exposure and kept its transparency, with only minimally altered color of the bronze, preserving visual authenticity. Importantly, the coating was also removable with ethanol under ultrasonic treatment, aligning with conservation principles of reversibility and minimal intervention. The claimed “labyrinth effect” and the strong adhesion were due to the Si–O–metal bonds, which impeded the penetration of corrosive ions, yielding suitable properties to consider the coating for long-term heritage bronze conservation and protection (Figure 6b).

Several papers have been published on protective coatings for bronze artworks in outdoor environment. For example, Pellis et al. [77] explored the possibility to modify commercial Paraloid^®^ B44, which degrades over time (its photo-oxidation is often considered an environmental concern), by incorporating nontoxic corrosion inhibitors and light stabilizers, for more sustainable and effective alternatives. Three corrosion inhibitors were compared, namely, the widely used benzotriazole (BTA) and the two alternatives 5-mercapto-1-phenyltetrazole (MPT) and 5-ethyl-1,3,4-thiadiazol-2-amine (AEDTA). Attention was given to how these three inhibitors behave in the presence of a bronze substrate since interaction with the metal can influence their retention and effectiveness. Among all, the inclusion of AEDTA, combined with light stabilizers, showed superior performance under several aspects; it remained within the coating and at the bronze interface, thus reducing leaching and enhancing long-term protection. These effects are believed to be a direct consequence of the presumed formation of Cu–inhibitor complexes, attenuating inhibitor release into the environment. In contrast, BTA and MPT were less stable and contributed to faster coating degradation unless they were stabilized with additional additives.

Regarding the ICOM preventive conservation criteria, some considerations need to be made for these two papers. The sol–gel fluoropolymer–silica hybrids [75] showed high transparency and stability but only partial reversibility, while safety and sustainability were considered in general terms rather than through detailed assessments (fluoropolymers are usually considered intrinsically not fully sustainable). The Paraloid^®^ B44-based coatings for outdoor bronze artworks [77] ensure transparency, improved stability, and enhanced safety through the replacement of hazardous BTA with less toxic inhibitors, with partial but promising reversibility. Importantly, the latter study also explicitly integrated sustainability by reducing environmental impact through nontoxic additives and light stabilizers, thus moving closer to fulfilling this criterion than many comparable works.

### Wood substrates

Wood substrates, when constituting part of artworks, face a wider range of threats due to their organic nature. Table 3 provides a summary of heritage wooden substrates, the coating base materials used for their protection, and the observed functional properties. Zouari et al. [78] explored two different coating materials (linseed oil and waterborne acrylic), used together with biocarbon (BC; produced by the pyrolysis of *Arundo donax* plant) doped with manganese oxide (BC–MnO_2_, used for formaldehyde removal), tested on beech (*Fagus sylvatica L.*) and oak (*Quercus robur*) substrates. The addition of BC–MnO_2_ particles increased hydrophobicity across all formulations, with contact angles ranging from 97° to 121°, and further improvements were observed after aging due to increased surface roughness. It also provided superior color stability and good formaldehyde removal, thus offering a passive, energy-efficient approach to improve indoor air quality while protecting wooden surfaces from environmental degradation.

**Table 3:** Coating materials used for heritage wooden substrates with their new features.

Application substrate(s)	Materials/composites	Feature	ICOM criteria (+1)^a^					Ref.
			Tr	St	Sa	Re	Su	

wood (beech and oak)	linseed oil or acrylic + biocarbon (from *Arundo donax*) doped with MnO_2_	hydrophobicity, color stability, formaldehyde removal	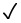	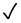	#	#	~	[78]
oak wood (*Quercus pubescens*)	Paraloid^®^ B72 + 1–4% silica dioxide nanoparticles	UV and moisture resistance, improved durability, color darkening	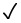	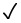	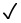	~	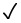	[79]
wood (heritage and construction use)	UV-curable acrylic-alkyd + vinylated alkyd oligomers + biocide (Polyphase R2085)	fast-curing, biocidal, mechanical strength, outdoor durability	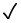	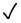	#	#	~	[80]
wood (oak, chestnut)	Dynasylan Sivo 121 + nano/submicrolignin (4% w/w)	superhydrophobicity, UV resistance, durability, rose-petal effect	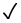	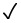	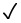	#	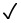	[81]

^a^Tr: optical transparency and non-intrusiveness (the coating should not alter appearance, color, gloss, or texture); St: chemical stability and durability (the coating is resistant to environmental agents like light, humidity, and pollutants); Sa: safety and ethical acceptability (no hazardous substances during coating application, and respect for cultural/historical significance); Re: reversibility or removability (the coating can be removed without damage, allowing future conservators to restore the object to its prior state); Su: sustainability (preference for environmentally responsible materials and processes, reducing the ecological footprint throughout the coating’s life cycle). 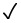: yes; ×: no; ~: partially; #: unclear.

Oak wood (*Quercus pubescens*) was also the focus of Mitani and colleagues [79]. They evaluated the surface modification of the wood using Paraloid^®^ B72 solutions with 1–4% silica dioxide nanoparticles to enhance durability and reduce moisture sensitivity (mostly to face the biotic and abiotic factors that usually lead to wood damage). The treatment improved dimensional stability, hydrophobicity, and resistance to artificial weathering, especially at higher nanoparticle concentrations. Color stability increased, but a slight darkening and higher surface roughness was spotted, which may limit use for outdoor applications. Overall, the Paraloid–silica nanoparticle coatings effectively enhanced UV, moisture, and biological resistance, offering promising potential for indoor preservation of heritage timber structures.

Also, bio-based solutions for wood protection coatings have been considered, many of which became increasingly efficient by the inclusion of specific additives to be used for targeted threats. For example, in case of wood, the attachment by fungal organisms must be effectively impeded, as demonstrated by Il’ina and colleagues [80]. A UV-curable, photo-reactive acrylic alkyd composition, enhanced with a biocide additive for protecting wooden surfaces against several fungi, was explored. The innovative formulation allows for the creation of thick, durable coatings (up to 80 µm) without sacrificing polymerization efficiency, as confirmed by Raman spectroscopy. Because of its UV-curing capability, the coating forms a hardened, protective layer within just 1–2 min. By incorporating vinylated alkyd oligomers (obtained from natural-derived raw materials), the coating’s overall mechanical performance was improved, including hardness, vapor permeability, and water repellency. A key feature of this system is its biocidal function; the addition of 0.24 wt % of commercial fungicidal agent (Polyphase R2085) effectively suppressed fungal growth from species such as *Aspergillus*, *Chaetomium*, *Trichoderma*, and *Penicillium*. These fungi are common culprits in the biodeterioration of timber, especially in outdoor environments. The cured coatings combine speed and strength with antimicrobial protection, making them suitable for preserving both new and historical wooden structures, also offering a compelling alternative to traditional solvent-based or slower-curing systems, meeting the modern demands of sustainability, efficiency, and long-term durability.

**Figure 7:**
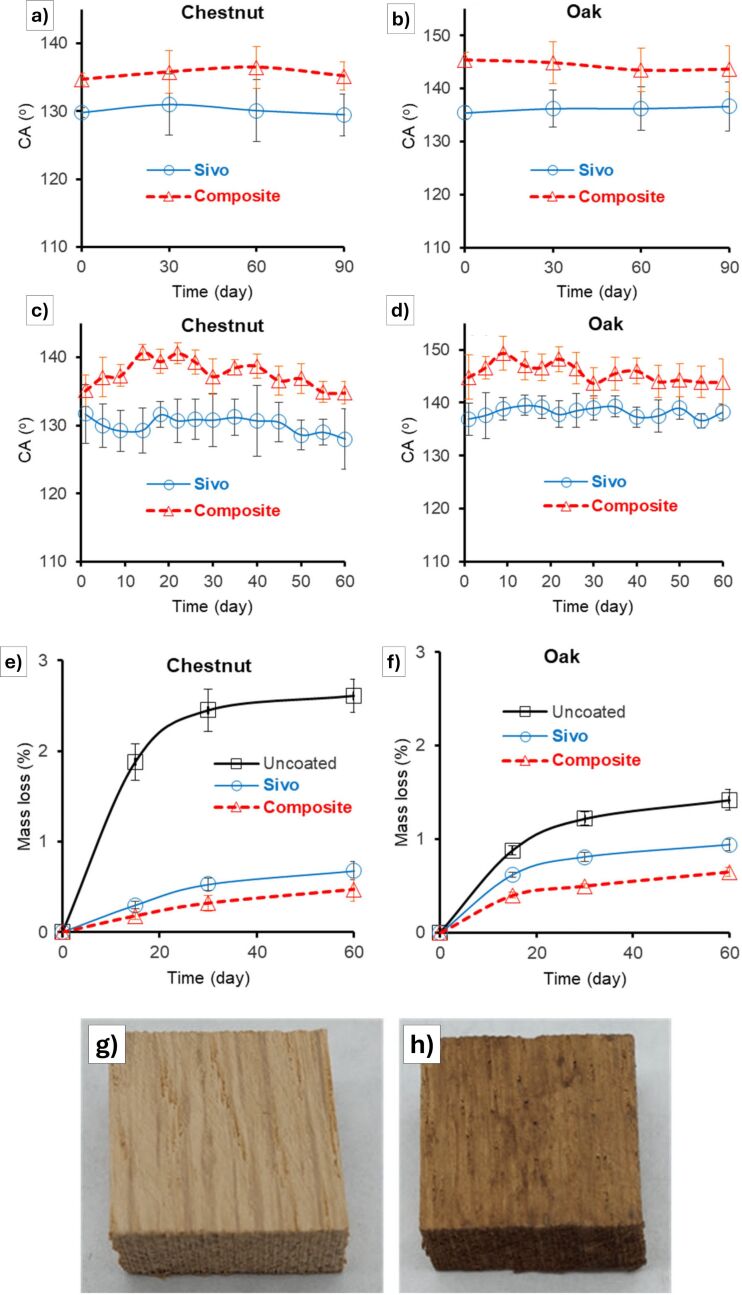
Contact angle as a function of the exposure time for wood samples kept (a, b) outdoors and (c, d) within the UV chamber. Results for wood samples coated with Sivo and wood samples coated with the selected composite (Sivo + 4.0% w/w NL) are shown. (e, f) Results of the biological durability soil burial test: mass loss (%) as a function of the period of time that wood samples remained buried in soil. Results for uncoated woods, woods coated with Sivo, and wood coated with the selected composite (Sivo + 4.0% w/w NL) are shown. The two photographs show oak specimens (g) before and (h) after the test. Figure 7 was reproduced from [81] (© 2025, M.M.M. Ramos et al., published by MDPI, distributed under the terms of the Creative Commons Attribution 4.0 International License, https://creativecommons.org/licenses/by/4.0).

Ramos et al. [81] described a sustainable approach for protecting wood (*Castanea sativa*, chestnut, and *Quercus pubescens*, oak) by developing a highly hydrophobic coating using environmentally friendly components. Their work focused on protection against the principal threat of water adsorption (which may cause swelling, warping, and structural weakening) by imparting long-lasting hydrophobicity, reducing water uptake, and maintaining dimensional and biological stability of wood, even under UV exposure and outdoor conditions (Figure 7). To do so, nano/sub-microlignin (NL), derived from beechwood via a one-step organosolv process, was used as a filler and incorporated into Dynasylan Sivo 121, a solvent-free silane system compatible with wood. The resulting composite coatings, particularly with 4% w/w NL, significantly enhanced water repellency on oak (water contact angle = 145°) and to a lesser extent on chestnut (water contact angle = 135°), with minimal impact on wood color. The coating maintained its hydrophobicity and mechanical durability after multiple tape peeling cycles and exposure to UV radiation and outdoor conditions (Figure 7a–d). Additionally, the coating demonstrated biological durability in soil burial tests, critical for preserving historic wood artifacts (Figure 7e–h). The rose-petal wetting effect (characterized by a high contact angle) [33,42] was observed, though further enhancement required a post-treatment for low surface energy.

The reported papers focused on wood substrates collectively highlight the efforts to align novel coating formulations with preventive conservation principles. All of these coatings achieved optical transparency and good long-term stability, while reversibility was only partially addressed as most systems involve cross-linked or hybrid chemistries that hinder complete removal. Safety considerations vary, with some reliance on biocides or fluorinated components, whereas the shift toward lignin- and resin-based formulations represents a safer and more acceptable route. Sustainability is explicitly tackled in the works employing natural resins and nanolignin [79,81], which emphasize renewable raw materials and low-toxicity systems, while the other studies [78,80] approach it only indirectly, through solvent-free processing or durability improvements rather than fully closed life-cycle considerations.

### Textile substrates

Just like wood, heritage textile materials are organic substrates since they are manufactured using plant- or animal-derived materials (i.e., cotton, wool, or silk); however, their protection against environmental threats is way less a consideration than that of the other materials. In Table 4, information on heritage textile substrates, applied coating materials, and resulting functional performance is shown.

**Table 4:** Coating materials used for heritage textile substrates, with their final new features.

Application substrate(s)	Materials/composites	Feature	ICOM criteria (+1)^a^					Ref.
			Tr	St	Sa	Re	Su	

gambiered Guangdong gauze (silk textile)	traditional mud-tannin coating from *Dioscorea cirrhosa* and river mud	UV resistance, antibacterial, visually darkened surface	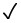	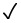	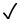	#	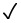	[82]
historic cotton textiles	TEOS (silica sol–gel) + dibutyltindiacetate (DBTA)	abrasion and wash resistance, thermal stability, flexible and transparent	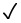	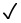	~	#	~	[83]

^a^Tr: optical transparency and non-intrusiveness (the coating should not alter appearance, color, gloss, or texture); St: chemical stability and durability (the coating is resistant to environmental agents like light, humidity, and pollutants); Sa: safety and ethical acceptability (no hazardous substances during coating application, and respect for cultural/historical significance); Re: reversibility or removability (the coating can be removed without damage, allowing future conservators to restore the object to its prior state); Su: sustainability (preference for environmentally responsible materials and processes, reducing the ecological footprint throughout the coating’s life cycle). 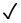: yes; ×: no; ~: partially; #: unclear.

Each culture has its own tradition in fabric manufacturing, and each culture has its own traditional method to protect such materials. One example is gambiered (gambier is a resinous substance obtained from the *Uncaria gambir* plant from Southeast Asia [84]) Guangdong gauze, a traditional Chinese silk fabric recognized as national intangible cultural heritage and distinguished by its exclusive use of pure plant and mineral dyes. The coating of this fabric is formed through a complex, manual dyeing and finishing process involving *Dioscorea cirrhosa* Lour extract (a plant source of tannins) and river mud, applied in multiple stages including steaming, boiling, sun-drying, and layering. The key functional components contributing to the fabric’s UV resistance are tannins, metal ions, humus, and fulvic acid. These interact with silk protein fibers to form a dark coating that absorbs UV and visible light, enhancing UV resistance and providing additional antibacterial properties. The authors of [82] reported the superior UV resistance of silk treated with this traditional method, but stated the evident difficulty of industrial scaling of this process. Several groups of scholars have investigated protection of historic textiles. Trovato et al. [83] reported the development of protective silica coatings, specifically designed for historic cotton textiles. They employed a sol–gel approach using the well-known TEOS as a silica precursor in two different concentrations, in the presence of dibutyltindiacetate (DBTA) as a catalyst, to promote polycondensation and enhance the coating’s performance; TEOS improved the thermal stability and washing resistance and DBTA enhanced the abrasion resistance, increasing with the number of layers applied. The important outcome is that, even upon application of multiple layers, the fabric’s flexibility and visual integrity was maintained.

In the papers reported here on heritage textile protection, good optical transparency and stability were achieved, ensuring that the coatings do not compromise textile appearance while improving resistance to UV, washing, or abrasion. Reversibility has not been explicitly addressed in either study, leaving this criterion unclear. Safety is well respected in the work on gambiered Guangdong gauze [82], which relies on natural polyphenols, but less so in the sol–gel silica approach that requires organotin catalysts [83]. Sustainability is a strength of the gauze study, which emphasizes natural dyes and bio-based additives, whereas in the sol–gel work it is only indirectly considered through durability improvements rather than a full life-cycle perspective.

### Superhydrophobicity and its applications in cultural heritage protection

**Figure 8:**
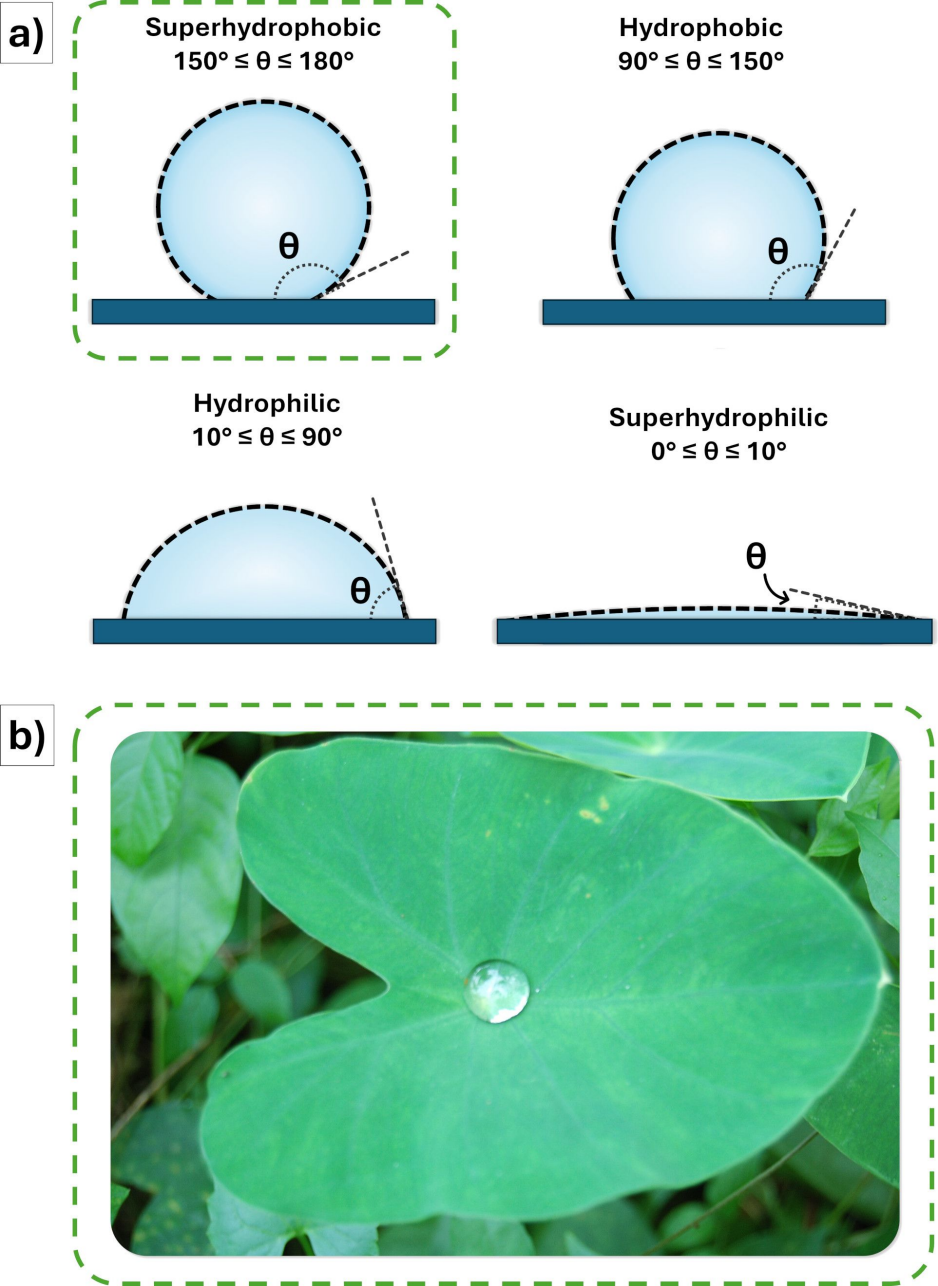
(a) Schematics of liquid droplets in contact with superhydrophobic, hydrophobic, hydrophilic, and superhydrophilic solid surfaces. (b) Real-life lotus-leaf effect.

Superhydrophobic coatings are used to prevent water-related damage. In order to fall into this definition, the contact angle of the water droplet on the studied surface should be equal or higher than 150° [33,42] (Figure 8a).

Superhydrophobicity has gained increasing attention since its first accurate analyses and observation in the late 1990s, when Barthlott and Neinhuis [85] first explained the water-repellent and self-cleaning properties of lotus plants (Figure 8b), ascribing these effects to epicuticular wax crystalloids, present in nano- and micrometric textures on the surface of its leaves [86]. In recent years, many studies have been carried out to fabricate superhydrophobic surfaces by using different strategies [33,42], whose most common are chemical vapor deposition, electrospinning, etching, and self-assembly. Moreover, the range of potential applications of superhydrophobicity is considerably wide; anti-biofouling, anti-corrosion, anti-bacterial, anti-icing, anti-fogging, and self-cleaning properties can be provided to surfaces by application of superhydrophobic coatings.

The main result of a water contact angle greater than 150° is that water drops roll off the surface, rather than wetting it. Regarding the origin of this natural “lotus effect”, the underlying mechanism involves a combination of surface roughness at the micro- and the nanoscale and low-surface-energy materials. The translation of this natural phenomenon into synthetic materials, better known as biomimetics, represents the main challenge in this field. It involves the creation of a structure with hierarchical roughness, combined with the application of hydrophobic substances, typically fluorinated compounds or silicone-based materials [33,42].

The application of superhydrophobic coatings in the context of cultural heritage preservation is a rapidly developing field; it encompasses a wide range of materials, including stone, wood, textiles, and metals, each presenting unique requirements for conservation (as explained in the previous section for coatings with other features) [87–88]. In general, the introduction of superhydrophobic coatings offers several benefits, like the protection against water damage (swelling, cracking, biological growth, or chemical reactions) [89], mitigation of urban pollution effects (reduced penetration of harmful pollutants and acids) [90], and self-cleaning properties (repelling dirt and contaminants, or even preserving from vandalism) [91], all aiming to prolong the lifespan of heritage materials.

A comprehensive study in this field was made by He and colleagues [40]. They focused on the production of a single superhydrophobic coating to be applied on different cultural heritage materials substrates including paper, wood, and fabric to be shielded from water, humidity, and UV damage. The coating combined the well-known and widely applied Paraloid^®^ B72 with 20 nm hydrophobic silica nanoparticles, dispersed in xylene, and applied via a one-step spray method. The optimal formulation (labeled BS2) included 2 wt % silica, balancing superhydrophobicity, transparency, and breathability. The BS2 coating achieved high water contact angles (up to 165°) and low sliding or roll-off angles (6°), indicating excellent water repellency. It retained superhydrophobic properties across a wide pH range (1–13), under high humidity, and after mechanical stress, such as sand impingement and simulated acid/alkali rain. Scanning electron microscopy (SEM) and FTIR analyses confirmed the uniform distribution and chemical integration of silica nanoparticles within the polymer matrix. UV–vis spectroscopy revealed improved UV blocking (only 19.46% transmittance vs 32.26% for pure B72), and the visual transparency remained above 72% in the visible range. Air permeability tests demonstrated that BS2 allowed limited moisture transmission while preventing water absorption. Even after prolonged exposure to hot humid environments, the coated materials resisted ink diffusion and color change, reducing the risk of moisture-driven aging. Durability tests showed that BS2 coatings maintained hydrophobicity over 30 days of outdoor exposure. Compared to uncoated or B72-only surfaces, BS2 offered superior resistance to UV-induced degradation and color yellowing. Moreover, the authors concluded that BS2 coating minimally altered the visual appearance of the substrate, making it a promising solution for non-invasive, breathable, and moisture-resistant protection of cultural relics.

In this section, other findings in this field achieved in the last five years are presented for heritage mineral-based (stone, concrete, and ceramics), metal-based (bronze and iron) and wood substrates.

### Superhydrophobic coatings for heritage mineral-based substrates

Several case studies have been published presenting practical implementations and highlighting the effectiveness of superhydrophobic coatings in the general field of cultural heritage preservation. In Table 5, specific cases of different mineral-based substrates, along with the corresponding coating materials and resulting properties, are presented.

**Table 5:** Superhydrophobic coating materials used for heritage mineral-based substrates with their new features.

Application substrate(s)	Materials/composites	Feature	ICOM criteria (+1)^a^					Ref.
			Tr	St	Sa	Re	Su	

marble, glass, wood, paper, etc.	TEOS + FAS^cb^	superhydrophobic, tested on multiple substrates	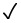	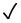	~	#	~	[92]
limestone	Silane + ZnO nanoparticles (NPs)	multifunctionality: superhydrophobic + photocatalytic + biocidal	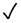	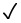	~	#	~	[93]
sandstone, granite, mortar, limestone	3D superhydrophobic agent + TiO_2_/ZnO heterostructures	focus on photocatalytic; NO degradation and transparency	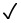	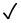	~	#	~	[94]
limestone and marble	CaCO_3_ NPs (dodecanoic acid-modified) + KSE 100^c^	enhanced durability tests (salt, freeze-thaw, UV)	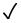	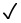	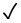	#	~	[95]
marble	Ca(OH)_2_ NPs + silane/siloxane	chemically compatible, compared with SiO_2_ coatings	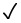	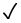	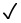	#	~	[96]
Alfas, Pentelic, and Carrara marble	sol–gel with TEOS + PDMS + SiO_2_ NPs	eco-friendly and crack-free nanocomposite	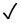	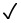	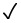	#	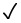	[97]
historic stone artifacts	oligo(ethylensuccinamide) + perfluoropolyether (PFPE)	focus on vapor permeability and magnetic resonance imaging (MRI) studies	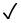	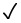	~	#	~	[98]
Stone	SiO_2_ + TiO_2_ + DTMS^d^	superhydrophobicity and UV resistance	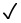	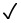	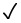	#	~	[99]
Concrete	SiO_2_ (30 nm) + epoxy resin (E-51) + HDTMS^e^, iron oxide pigments (red, yellow, blue, green)	durable multicolored superhydrophobic coating with; resistant to abrasion, sand impact, extreme weather, and chemical attack (acid/alkali)	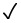	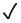	~	#	~	[100]

^a^Tr: optical transparency and non-intrusiveness (the coating should not alter appearance, color, gloss, or texture); St: chemical stability and durability (the coating is resistant to environmental agents like light, humidity, and pollutants); Sa: safety and ethical acceptability (no hazardous substances during coating application, and respect for cultural/historical significance); Re: reversibility or removability (the coating can be removed without damage, allowing future conservators to restore the object to its prior state); Su: sustainability (preference for environmentally responsible materials and processes, reducing the ecological footprint throughout the coating’s life cycle). 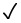: yes; ×: no; ~: partially; #: unclear; ^b^FAS: 1*H*,1*H*,2*H*,2*H*-perfluorooctyltriethoxysilane; ^c^KSE: commercial stone strengthener; ^d^DTMS: dodecyltrimethoxysilane; ^e^HDTMS: hexadecyltrimethoxysilane.

For historical monuments and buildings, Ray et al. [101] described the production of a quasi-superhydrophobic coating starting from silica nanoparticles, obtained by hydrolysis of TEOS in ethanol under alkaline conditions, further treated with hexadecyltrimethoxysilane (HDTMS) solution to enhance hydrophobicity. Both layers (silica nanoparticles and HDTMS solution) were applied by spray coating. The coated surfaces showed high water contact angles (121°–135°) and low sliding or roll-off angles (9°–22°), demonstrating strong water repellency and self-cleaning behavior, even after accelerated weathering and exposure to salt water, proving the potential for real-world applications.

Other recent advances in the protection of cultural heritage stone materials have focused on the development of multifunctional coatings that combine superhydrophobicity with additional protective features, using hybrid nanocomposite formulations. A unifying strategy across these studies is the integration of silicon-based matrices (such as TEOS, silanes, and siloxanes) with nanoparticles including ZnO, TiO_2_, SiO_2_, CaCO_3_, and Ca(OH)_2_. These materials are chosen for their ability to create micro/nanoscale surface roughness and reduce surface energy, which together enables water repellency characterized by high static contact angles (>150°) and low sliding or roll-off angles (<10°). For example, the TEOS + FAS system in [92] achieved extreme values with contact angles above 170° and sliding or roll-off angles below 5°, confirming its superhydrophobic performance. Most formulations are applied via simple methods like spray coating and are evaluated for key conservation parameters such as water resistance, color stability, vapor permeability, and substrate compatibility.

**Figure 9:**
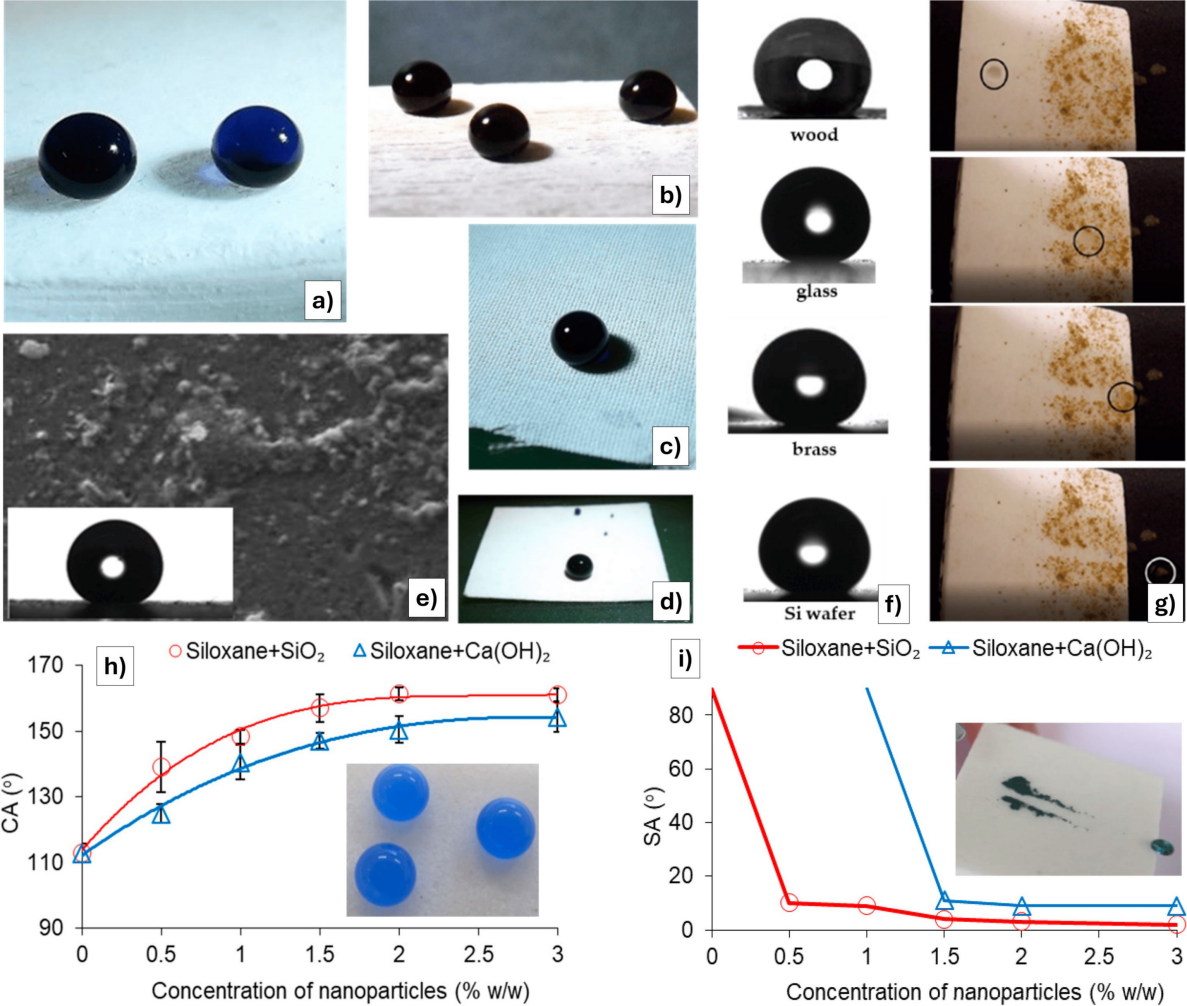
Photographs of water drops on coated (a) marble, (b) wood, (c) silk, and (d) brass. (e) SEM image of the surface of coated marble and a side-view photograph of a water drop. (f) Side-view photographs of water drops on various coated substrates. (g) Easy/self-cleaning demonstrated in a series of snapshots from top to bottom. The motion of a water drop is captured by the circles. Figure 9a–g was adapted from [92] (© 2020, F.G. Adamopolous et al., published by MDPI, distributed under the terms of the Creative Commons Attribution 4.0 International License, https://creativecommons.org/licenses/by/4.0). (h) Static contact angle (CA) and (i) sliding or roll-off angle (SA) vs the nanoparticle concentration. Data points were (h) fitted with polynomial functions and (i) connected with lines to guide the eye. Coatings prepared using <0.5% w/w SiO_2_ and <1.5% w/w Ca(OH)_2_ were pinned on the surfaces; therefore, they correspond to a theoretical SA of 90°. Photographs showing (h) resting drops and (i) the self-cleaning process were taken for marble specimens that were coated with superhydrophobic and water repellent composite coatings. The latter were prepared using siloxane and 3% w/w Ca(OH)_2_ nanoparticles. Figure 9h,i were adapted from [96] (© 2020, A. Chatzigrigoriou et al., published by MDPI, distributed under the terms of the Creative Commons Attribution 4.0 International License, https://creativecommons.org/licenses/by/4.0).

Despite the variation in materials and target substrates (e.g., limestone, marble, sandstone, or mortar), all studies in the field share a focus on maintaining the visual and physical integrity of the treated surfaces (Figure 9a–g). Reversibility (or minimal penetration) is required in line with conservation ethics. While some scholars prioritize environmental resilience and aging resistance (e.g., coatings tested under simulated rain, thermal cycling, or solar irradiation), others highlight multisubstrate applicability, extending protective technologies to non-stone materials like paper, glass, or fabric. The research field underscores the evolution of conservation from purely hydrophobic barriers to smart, multifunctional, nanostructured systems capable of addressing the complex and synergistic degradation processes the heritage materials have to face in the modern era with changing environmental conditions.

Several studies have aimed to enhance basic hydrophobicity with additional functionalities tailored to specific conservation needs. For instance, ZnO and TiO_2_-based coatings [93–94] introduce photocatalytic and biocidal properties, enabling not just passive water repellency but active degradation of pollutants and microbial inhibition. Calcium-based nanoparticles like CaCO_3_ and Ca(OH)_2_ [95–96] are chemically compatible with calcareous stones and offer excellent durability under stress conditions like freeze–thaw cycles, salt crystallization, and UV exposure. Other systems integrate hydrophobic polymers such as PDMS or perfluoropolyethers [97–98], producing eco-friendly coatings that preserve breathability and avoid toxic solvents, important for applications on heritage artifacts.

Dodecyltrimethoxysilane (DTMS) is widely used in cultural heritage conservation but suffers from poor UV stability and moderate durability. Peng et al. [99] focused on enhancing the coating performances by incorporating TiO_2_ (to improve thermal properties) and SiO_2_ (to provide roughness to enhance superhydrophobicity) nanoparticles. The coating with both fillers achieved water contact angles above 152° and roll-off angles below 10°. Water absorption tests showed over 92% reduction compared to untreated stone, and vapor permeability was maintained above 82%, preserving the stone’s breathability. Regarding the durability, the coating showed enhanced thermal stability, with decomposition temperatures higher for those with fillers. Interestingly, UV tests revealed that TiO_2_ alone reduced hydrophobicity under irradiation due to photocatalysis, but the inclusion of SiO_2_ mitigated this effect, preserving performance. Chemical stability was also improved, especially under neutral and alkaline conditions.

**Figure 10:**
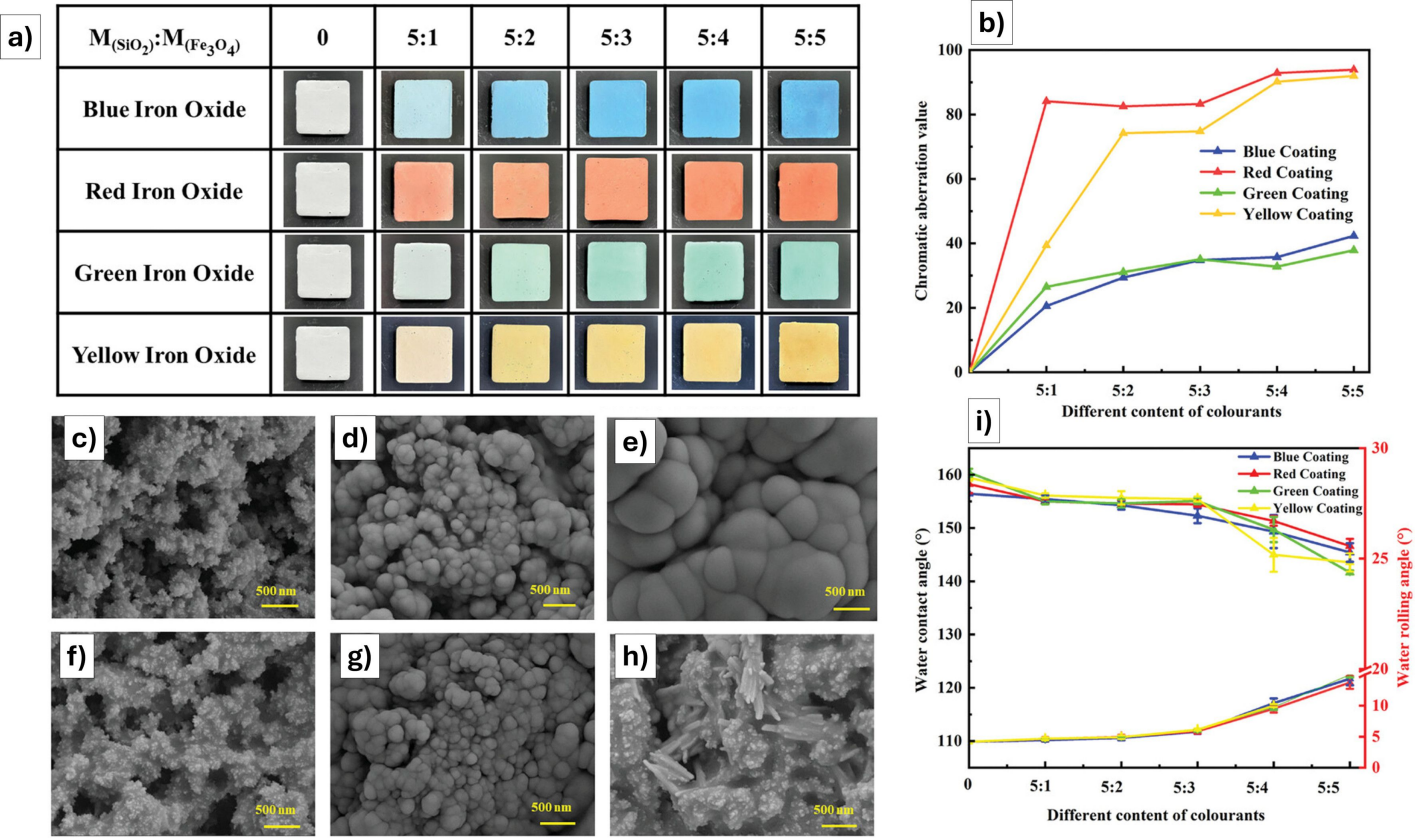
Effect of different colorants in colored superhydrophobic coatings. (a) Effect of varying ratios of colorants on superhydrophobic coating coloration. (b) Relationship between the total color difference (Δ*E*) value and dye content in the colored superhydrophobic concrete coatings. (c–e) SEM images of superhydrophobic concrete with different proportions of green colorant. (f–h) SEM images of blue, red, and yellow superhydrophobic coatings, respectively. (i) Influence of varying dye content on the contact angle and rolling angle of superhydrophobic concrete coatings. Figure 10 was adapted from [100], (© 2025 X. Shi et al., published by Wiley-VCH GmbH, distributed under the terms of the Creative Commons Attribution 4.0 International License, https://creativecommons.org/licenses/by/4.0).

Regarding heritage concrete, introducing superhydrophobicity on its surface is in order to overcome issues related to water permeability, environmental degradation, connected to concrete’s intrinsic porosity and hydrophilicity, which make it prone to damage from corrosion, erosion, acid rain actions, and in some cases also from ice formation [88] especially in outdoor environments. In [100], together with these more practical issues, the authors wanted to solve the limited visual design options when choosing concrete materials (Figure 10a,b). Shi et al. introduced a simple and effective method for creating durable, multicolored superhydrophobic coatings for concrete surfaces. They developed a coating applicable through a one-step spraying technique that combines silica nanoparticles (30 nm), epoxy resin (E-51), hexadecyltrimethoxysilane (HDTMS), and iron oxide-based pigments (red, yellow, blue, and green). The actual preparation involved dispersing SiO_2_ in ethanol via ultrasonication, then mixing in HDTMS and an epoxy hardener (tetraethylenepentamine, TEPA) solution under continuous stirring. Iron oxide dyes were added for coloration, and the mixture was ultrasonicated again to produce a uniform sprayable solution. The synergy between HDTMS and SiO_2_ imparts superhydrophobicity by reducing surface energy and introducing micro/nanoscale roughness (Figure 10c–i), while the epoxy resin ensures strong adhesion and mechanical robustness through hydrogen bonding with the concrete surface. The resulting coating yielded a high water contact angle (156° ± 1°) and a low sliding or roll-off angle (5° ± 1°), confirming excellent water repellency (Figure 10j), which was maintained even after abrasion, sand impact, and after exposition to weather conditions including sunlight, rainfall, and wind. It also resisted chemical damage from acids and alkalis.

A detailed analysis of these papers, following the ICOM criteria, is required. Optical transparency is largely respected, with small or negligible chromatic changes reported for stone substrates in hybrid systems (TEOS/CaCO_3_ [95] and siloxane/Ca(OH)_2_ [96]), and deliberate loss of original color only where colorants are intrinsic to the formulation for concrete [100]. Stability is consistently strong; superhydrophobicity and water repellence are retained after capillary uptake tests, UV exposure, freeze–thaw and salt attack, and simulated rain/peeling in several works [92,95,99]. By contrast, reversibility remains unclear across the set; none of the papers provides a removal protocol demonstrating safe return to the prior state, even where low-*M*_W_ or non-crosslinking polymers are used. Safety is generally addressed implicitly via substrate compatibility and benign solvents (e.g., aqueous emulsions; fatty-acid-modified CaCO_3_; “eco-friendly” sol–gel). Yet, explicit toxicological assessment is rare; systems relying on ZnO biocidal action or fluorinated moieties are particularly flagged as needing caution despite performance advantages [93,98].

Concerning sustainability, several formulations are reported regarding material compatibility and benign processing. Calcium-based fillers (Ca(OH)_2_/CaCO_3_) are chemically compatible with calcareous stones and can be prepared/used in aqueous media, reducing volatile organic compounds (VOCs) and favoring future re-treatability. A “green strategy” TEOS–SiO_2_–PDMS sol–gel explicitly emphasizes nontoxic, eco-friendly, low-energy synthesis while delivering superhydrophobicity, indicating a promising path for cradle-to-application impact reduction [97]. Other sustainability levers are benefits during use; non-wetting, photocatalytic, or biocidal functionalities can reduce water ingress, cleaning frequency, and detergent use (e.g., siloxane–ZnO composites that combine lotus-effect rolling, photocatalytic self-cleaning, and antibacterial action), potentially lowering maintenance emissions over a monument’s life [93–94]. However, gaps remain. First, none of the studies provides a cradle-to-grave life-cycle assessment (LCA) or end-of-life pathway; nanoparticle release (abrasion/erosion) and the fate of additives are not quantified. Second, fluorinated components (TEOS–FAS; perfluoropolyether-grafted oligoamides) enhance performance but raise persistence/bioaccumulation concerns acknowledged by the authors themselves for PFPE derivatives, even when “environmentally friendly solvents” are used [98]. Third, epoxy-based binders for concrete provide durability yet can entail petrochemical feedstocks and amine hardeners, with limited discussion of VOCs, worker exposure, or de-coating [100]. Overall, the corpus demonstrates real progress toward durable, compatible, and often transparent protections; sustainability is partially addressed via compatibility, solvent choice, and reduced maintenance demand, but rigorous LCA, removability strategies, nanoparticle release metrics, and designs free of per- and polyfluoroalkyl substances (PFAS) are the next key steps for alignment with conservation ethics and environmental responsibility.

### Superhydrophobic coatings for heritage metal substrates

Recent developments in the protection of metal-based cultural heritage by means of superhydrophobic coatings have also embraced the potential multifunctionality of materials, aiming to prevent corrosion, to preserve aesthetic qualities, and to extend the service life under exposure to diverse environmental threats. In Table 6, information on heritage metal-based substrates, applied coating materials, and resulting functional performance, in particular superhydrophobicity, is shown. The most commonly adopted solution consists in formulating advanced hybrid coatings that combine low-surface-energy agents with micro- and nanostructured fillers to achieve superhydrophobicity, ensuring self-cleaning and anti-wetting properties. These coatings go beyond passive water repellency to actively protect against corrosion, UV degradation, and environmental pollutants.

**Table 6:** Superhydrophobic coating materials used for heritage metal-based substrates with their new features.

Application substrate(s)	Materials/composite system	Feature	ICOM criteria (+1)^a^					Ref.
			Tr	St	Sa	Re	Su	

bronze	FEVE^b^ polymer + MOF-derived NiCo_2_O_4_ nanocapsules + Ti_3_C_2_Tx MXene nanosheets	multifunctional coating with UV resistance, self-cleaning, and high corrosion protection	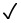	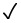	~	~	~	[102]
bronze	PFDTES + Ti_3_C_2_Tx MXene + TiO_2_ NPs	superhydrophobic with strong barrier effect, high weathering stability, and abrasion/UV resistance	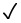	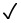	~	#	~	[103]
bronze, iron, pottery, sandstone, ivory, lacquer	nano-SiO_2_ + perfluorodecyltrimethoxysilane (via ultrasound probe)	non-whitening coating, highly portable, excellent for outdoor archaeological use	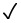	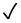	~	#	~	[89]
steel	PU^c^ + SiO_2_ nanoparticles + HDTMS	superhydrophobic, self-cleaning, anti-fogging, anti-corrosion	~	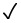	~	#	~	[104]

^a^Tr: optical transparency and non-intrusiveness (the coating should not alter appearance, color, gloss, or texture); St: chemical stability and durability (the coating is resistant to environmental agents like light, humidity, and pollutants); Sa: safety and ethical acceptability (no hazardous substances during coating application, and respect for cultural/historical significance); Re: reversibility or removability (the coating can be removed without damage, allowing future conservators to restore the object to its prior state); Su: sustainability (preference for environmentally responsible materials and processes, reducing the ecological footprint throughout the coating’s life cycle). 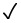: yes; ×: no; ~: partially; #: unclear; ^b^FEVE: fluoroethylene vinyl ether; ^c^PU: polyurethane.

**Figure 11:**
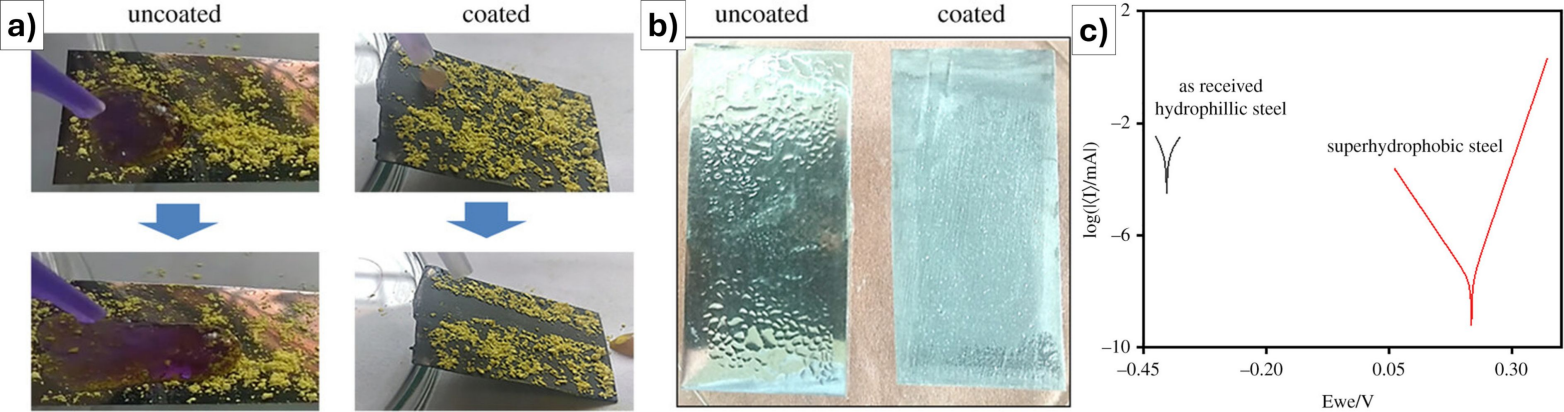
(a) Optical images of uncoated and coated samples while the self-cleaning test is carried out. (b) Optical images of uncoated and coated samples after exposure to water vapor. The uncoated sample shows a large number of water droplets, whereas the coated sample shows a much smaller number of water droplets. (c) Potentiodynamic polarization curve of uncoated and coated steel in 3.5 wt % of NaCl solution. Figure 11 was adapted with permission of The Royal Society of Chemistry, from [104] (“Development of polyurethane-based superhydrophobic coatings on steel surfaces” by M. K. Meena et al., *Phil. Trans. R. Soc. A*., vol. 378, issue 2167, © 2020); permission conveyed through Copyright Clearance Center, Inc. This content is not subject to CC BY 4.0.

In Wu et al. [102], a complex organic–inorganic hybrid system was synthesized using fluoroethylene vinyl ether (FEVE) as a matrix, reinforced with MOF-derived NiCo_2_O_4_ nanocapsules and Ti_3_C_2_T*_x_* MXene nanosheets. This multifunctional coating demonstrated excellent electrochemical corrosion resistance, maintained its superhydrophobicity (water contact angle ≈ 152.8°) even after UV aging, and did not alter the visual appearance of the bronze (Figure 11a–g). The superior performance was attributed to a “labyrinth effect” created by the hierarchical structure, which blocked the diffusion of corrosive agents and enhanced structural stability under harsh conditions. Additionally, this coating offered UV protection and self-cleaning ability, showcasing its comprehensive functionality for long-term outdoor preservation.

The same group introduced another superhydrophobic organic–inorganic composite based on PFDTES, Ti_3_C_2_T*_x_* MXene nanosheets, and TiO_2_ nanoparticles [103]. The fluorinated silane (PFDTES) contributed to ultralow surface energy, while Ti_3_C_2_T*_x_* and TiO_2_ provided physical barrier effects and UV/abrasion resistance. The coating yielded a water contact angle of 153° and formed a robust hydrophobic layer that resisted corrosion even after immersion in 3.5 wt % NaCl for seven days. Electrochemical tests confirmed significant reductions in corrosion current and increased impedance, demonstrating the effectiveness of the barrier network in protecting bronze. Moreover, this coating retained transparency and did not alter the original bronze appearance, satisfying critical conservation criteria. Both studies used Ti_3_C_2_T*_x_* MXene to enhance both physical protection and interface compatibility within the hybrid matrices.

Guo et al. [89] dealt with a frequent challenge in cultural heritage coatings (i.e., surface whitening) by developing a non-whitening superhydrophobic system composed of nano-SiO_2_ and perfluorodecyltrimethoxysilane, applied with an ultrasound probe. This method preserved the nanoscale morphology of SiO_2_, ensuring the formation of a porous hierarchical structure while avoiding visible residue on substrates, which is critical for maintaining fine surface details like inscriptions or patina. The coating exhibited exceptional dynamic wetting properties (water contact angle = 154°, roll-off angle = 1.7°) and resisted extreme physical and chemical stress, including high temperatures (up to 150 °C), prolonged UV exposure, acid/alkali immersion, and sand impact. Its broad applicability to diverse materials (bronze, iron, pottery, sandstone, ivory, and lacquer) combined with field-ready portability, makes it particularly suitable for in situ conservation in archaeological and museum contexts. This work, together with [103], exploited the inclusion of fluorinated silanes to further ensure ultralow surface energy and hydrophobic endurance under environmental stress.

Another approach was considered by Cremaldi and Bhushan [105] and Meena and colleagues [104]. The superhydrophobic coating was developed for steel surfaces, to address common industrial issues such as corrosion, water accumulation, and surface fouling (easily relatable to those of heritage steel). The mixture, composed of polyurethane (PU, resin material used in several application fields [106–108]), SiO_2_ nanoparticles, and HDTMS was applied on a pre-etched steel surface, by means of three steps of spin-coating, followed by curing at 100 °C. The coating demonstrated a static water contact angle of 165° and a roll-off angle of 4°, confirming excellent superhydrophobicity, having microstructures and functional materials responsible for water repellency (Figure 11a). The coating maintained its performance after thermal exposure up to 230 °C and under mechanical stress such as water jet impact, bending, and sand abrasion, though excessive abrasion did reduce effectiveness. Highly acidic or alkaline environments resulted in a degradation of the coating, but under near-neutral conditions the coating remained stable for weeks. Self-cleaning and anti-fogging properties were observed, with coated surfaces shedding water and dust effectively, also resisting condensation (Figure 11b). Corrosion resistance was significantly improved, with the corrosion current density dropping from 0.825 µA/cm^2^ (uncoated) to 0.0002 µA/cm^2^ (coated) (Figure 11c).

To summarize, these works illustrate a clear trend in the field of metal heritage objects toward coatings that are not only superhydrophobic but also smart and multifunctional, aiming to combine corrosion inhibition, UV stability, aesthetic compatibility, and ease of application. These properties make them highly promising for the protection of both indoor museum pieces and outdoor archaeological bronzes. As environmental pressures and conservation demand increase, such coatings represent a critical evolution in material science for cultural heritage preservation.

In terms of ICOM criteria, above four studies emphasize the development of multifunctional superhydrophobic coatings for metals and heritage materials, with varying alignment to preventive conservation principles. Optical transparency is generally well maintained in the bronze-focused coatings [102–103], including the non-whitening SiO_2_/PFDMS system [89], although the polyurethane–SiO_2_–HDTMS steel coating [104] does not prioritize transparency. Stability is a strength across all cases, supported by weathering, UV, abrasion, and electrochemical tests that confirm long-term protective performance. Reversibility remains problematic, rarely addressed beyond general claims of removability, leaving it largely unclear or only partially fulfilled. While reliance on fluorinated compounds and polyurethane precursors raises environmental and health concerns, efforts to improve compatibility and minimize whitening contribute to substrate integrity.

On sustainability, the approaches remain only partially aligned with conservation ethics. The FEVE and PFDTES-based hybrids [102–103] depend on fluorocarbon chemistry, offering excellent durability but at the cost of persistence and bioaccumulation risks. The non-whitening PFDMS/SiO_2_ coating [89] introduces operational benefits by preventing aesthetic damage and enabling scalable in situ use, yet it still relies on fluorosilanes. The polyurethane-based system [104] is used as bioinspired but is fundamentally petrochemical and solvent-intensive, with limited end-of-life considerations. Overall, while these coatings achieve high stability and transparency, their sustainability remains constrained by reliance on fluorinated or petrochemical components, with durability often used as a proxy for ecological acceptability rather than a full assessment of environmental footprint.

### Superhydrophobic coatings for heritage wood substrates

**Figure 12:**
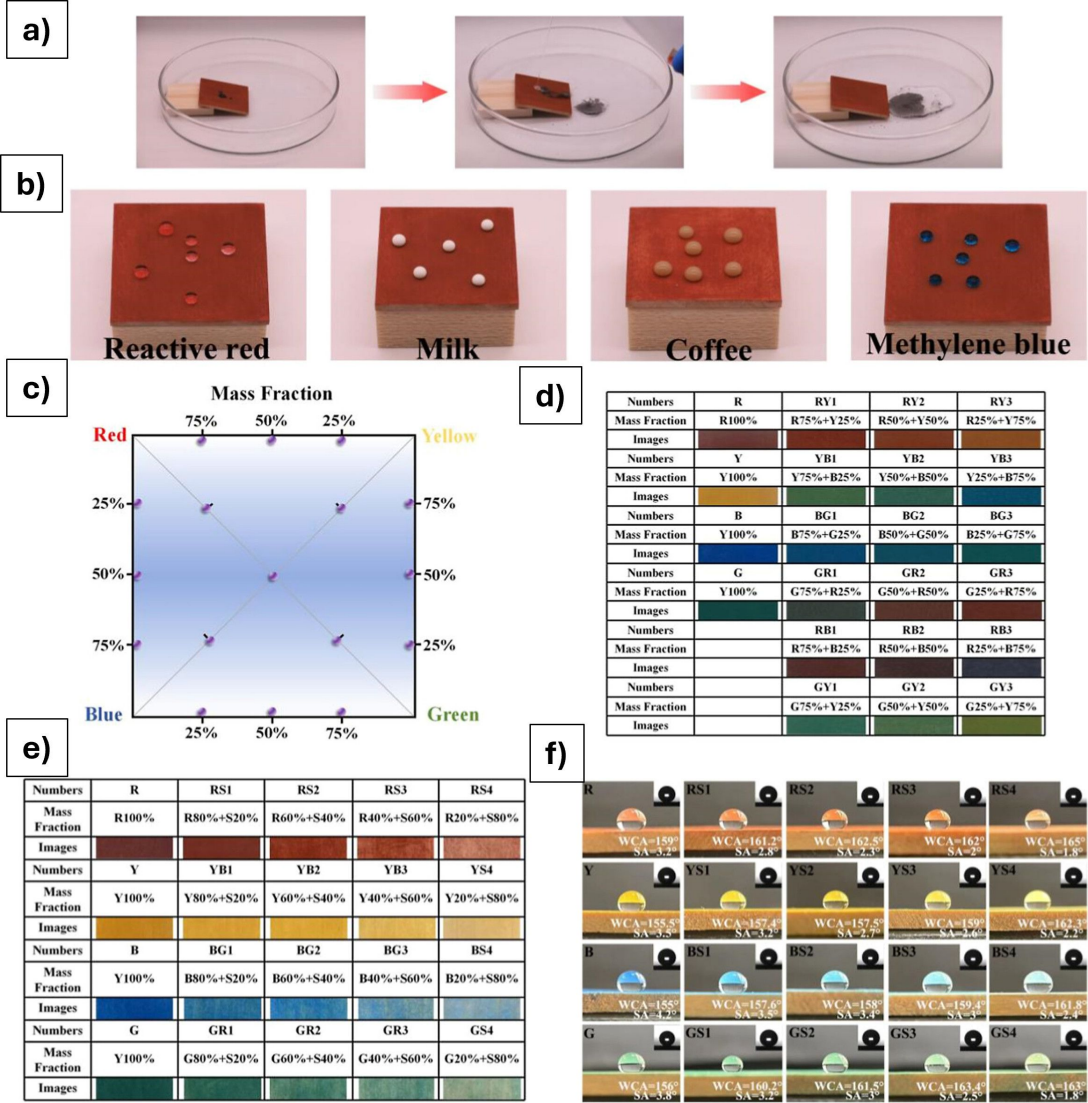
Analysis of antifouling and self-cleaning properties of colored superhydrophobic coatings and coating color diversity. (a) Rsw selfcleaning test. (b) Rsw antipollution test. (c) Four modified iron oxide powders proportionally mixed with concentration squares. (d) 22-color iron oxide powder corresponding to (c). (e) Color gradient photographs of a proportionally mixed mixture of hydrophobic SiO_2_-modified iron oxide powders. (f) Contact angle images of each gradient of color superhydrophobic wood corresponding to (e). Figure 12 was adapted with permission from [109], Copyright 2024 American Chemical Society. This content is not subject to CC BY 4.0.

**Figure 13:**
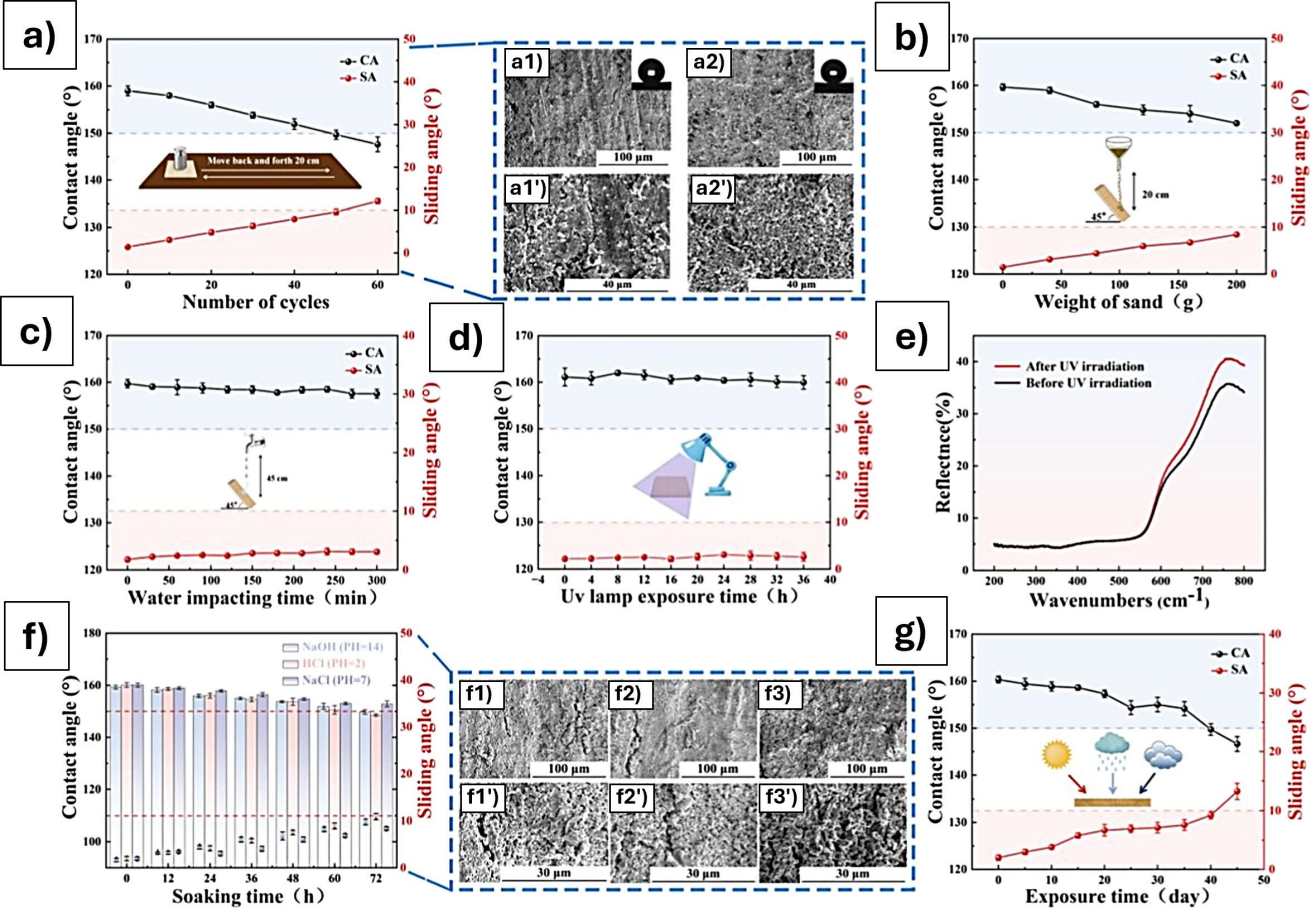
Mechanical−chemical durability testing of colored superhydrophobic coatings. (a) Sandpaper abrasion test, (a1, a1′) SEM image after sandpaper abrasion, and (a2, a2′) SEM image after repair. (b) Impact resistance test. (c) Water impact test. (d) UV lamp irradiation test. (e) Reflectance spectrograms before and after UV lamp irradiation. (f) Acid and alkali corrosion solution test, (f1, f1′) Rsw strong alkali solution immersion for 72 h SEM image, (f2, f2′) Rsw strong acid solution immersion for 72 h SEM image, and (f3, f3’) Rsw corrosive solution immersion for 72 h SEM image. (g) Weather resistance test. Figure 13 was adapted with permission from [109], Copyright 2024 American Chemical Society. This content is not subject to CC BY 4.0.

Heritage wooden objects are, in number, less than those made of metal or mineral-based materials. Thus, in literature, few works are devoted to solutions. However, recent research has offered promising solutions for enhancing the performance of heritage wooden materials with superhydrophobic, self-cleaning, and durable surface coatings. In one study, Yao et al. [109] developed colored superhydrophobic coatings for wood by combining iron oxide-based pigments modified with PDMS, hydrophobic SiO_2_ nanoparticles, and epoxy resin (EP). Applied to treated poplar wood via a “sand-in” method involving primer spraying, sanding pigment powder, and PDMS–EP topcoating followed by heat curing, the coatings achieved micro/nanostructured surfaces and contact angles up to 165°, demonstrating strong water repellency (Figure 12a–f). These coatings resisted mechanical abrasion, UV exposure, and chemical degradation. Their superhydrophobicity was restorable with a simple topcoat reapplication, and their self-cleaning properties were effective even against particulate and liquid contaminants (Figure 13a–g).

In a different approach, on the same poplar wood substrate, Wang et al. [110] pursued a more sustainable route using natural components (a combination of ethyl cellulose (EC), stearic acid (SA), and shellac) to form a self-assembled bilayer coating. EC/shellac first filled the wood pores, forming a strong adhesive substrate, followed by an EC/SA treatment that introduced hierarchical roughness through alkyl group-driven nanosheet assembly. The resulting surface had a water contact angle of 162° and a sliding or roll-off angle of 2.7°, with significantly reduced moisture absorption. The micro/nanostructured coating, supported by hydrogen bonding, proved resistant to UV aging, mechanical wear, and chemical exposure. Self-healing was observed through simple drying after surface damage or immersion, while the overall system preserved stability and hydrophobicity over time. Both studies underscore distinct yet effective pathways (the first was synthetic, the second was natural) toward multifunctional wood surface coatings with enhanced durability, mechanical resilience, and water resistance.

These two papers highlight different strategies for producing durable superhydrophobic coatings on wood with relevance to conservation practices (Table 7). The colored PDMS/epoxy–iron oxide coatings [109] achieve high stability against abrasion, UV, and chemical exposure while providing decorative color options, though they inevitably alter optical appearance and offer no clear indication of reversibility. Safety and sustainability are only partly addressed as the former system relies on petrochemical epoxy resins and modified oxides, with claims of durability serving as the main ecological argument. In contrast, the latter ethyl cellulose/shellac/stearic acid coating [110] demonstrates strong alignment with conservation criteria: It maintains transparency of the wood surface, delivers excellent chemical and mechanical stability, and uses entirely bio-based, nontoxic components that ensure safety. Sustainability is a central strength of this work, since it combines renewable raw materials with a mild fabrication process, achieving a balance of durability, self-healing capacity, and ecological responsibility.

**Table 7:** Superhydrophobic coating materials used for heritage wood-based substrates with their new features.

Application substrate(s)	Materials/composite system	Feature	ICOM criteria (+1)^a^					Ref.
			Tr	St	Sa	Re	Su	

poplar wood (representing building and decorative wood)	PDMS-modified iron oxide pigments, hydrophobic SiO_2_, epoxy resin	colored coatings; abrasion resistance; self-repair via topcoat; pigment customizability	~	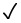	~	#	~	[109]
poplar wood	ethyl cellulose (EC), stearic acid (SA), shellac	natural components; bilayer self-assembly; self-healing by drying; environmentally friendly	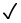	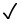	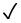	#	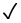	[110]

^a^Tr: optical transparency and non-intrusiveness (the coating should not alter appearance, color, gloss, or texture); St: chemical stability and durability (the coating is resistant to environmental agents like light, humidity, and pollutants); Sa: safety and ethical acceptability (no hazardous substances during coating application, and respect for cultural/historical significance); Re: reversibility or removability (the coating can be removed without damage, allowing future conservators to restore the object to its prior state); Su: sustainability (preference for environmentally responsible materials and processes, reducing the ecological footprint throughout the coating’s life cycle). 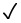: yes; ×: no; ~: partially.

### New developments: superoleophobic coatings

In addition to the nature-inspired superhydrophobic effect, particularly the abovementioned “Lotus effect” [33,42,111], there is another similar effect aimed to be reproduced for cultural heritage objects protection (in particular against threats like graffiti for which oil-based spray paintings are used), that is, superoleophobicity [33,41–42,112]. Its characteristics have the same definition as the superhydrophobic effect (contact angle higher than 150° and a sliding or roll-off angle lower than 10°), but considering oil drops, instead of water. Just like superhydropobic coatings, these coatings rely on surface roughness and chemical modifications to achieve extreme repellency, by means of materials, such as fluoropolymers, silica nanoparticles, fluorinated or silicone-based compounds, which create surfaces that drastically reduce oil adhesion, or that repel oils and non-polar liquids.

These coatings are already widely applied in several fields, in particular in building protection. For example, Cao et al. [113] studied a superamphiphobic, self-cleaning surface for outdoor application. They exploited the different surface porosity of two selected stones, Pietra Serena (low porosity) and Lecce stone (high porosity), for the tests. (3-aminopropyl)trimethoxysilane (APTMS) and acyl fluoride-functionalized perfluoroether (AF-PFE) reacted in nitrogen atmosphere at room temperature, and the coating material (Si-PFE) was obtained. It was dispersed in 2-propanol to be then applied on the stone surfaces by a “wet-on-wet” method. The final Si-PFE coating imparted superamphiphobic properties to stone surfaces, with both water and oil contact angles exceeding 150° (in some cases even over 160°), and low contact angle hysteresis, indicating strong repellence and effective self-cleaning. The surfaces remained superhydrophobic and oleophobic even after prolonged exposure to water, acidic (pH 2.9) and alkaline (pH 14) environments. The coating exhibited excellent mechanical durability, retaining its properties after 40 cycles of tape-peeling and sandpaper abrasion due to its integration within the stone’s porous microstructure, making it a robust and versatile solution for protecting porous building materials in harsh environments. For these reasons, these kinds of coatings have shown considerable promise in protecting heritage materials from both environmental wear and malicious vandalism [114].

**Figure 14:**
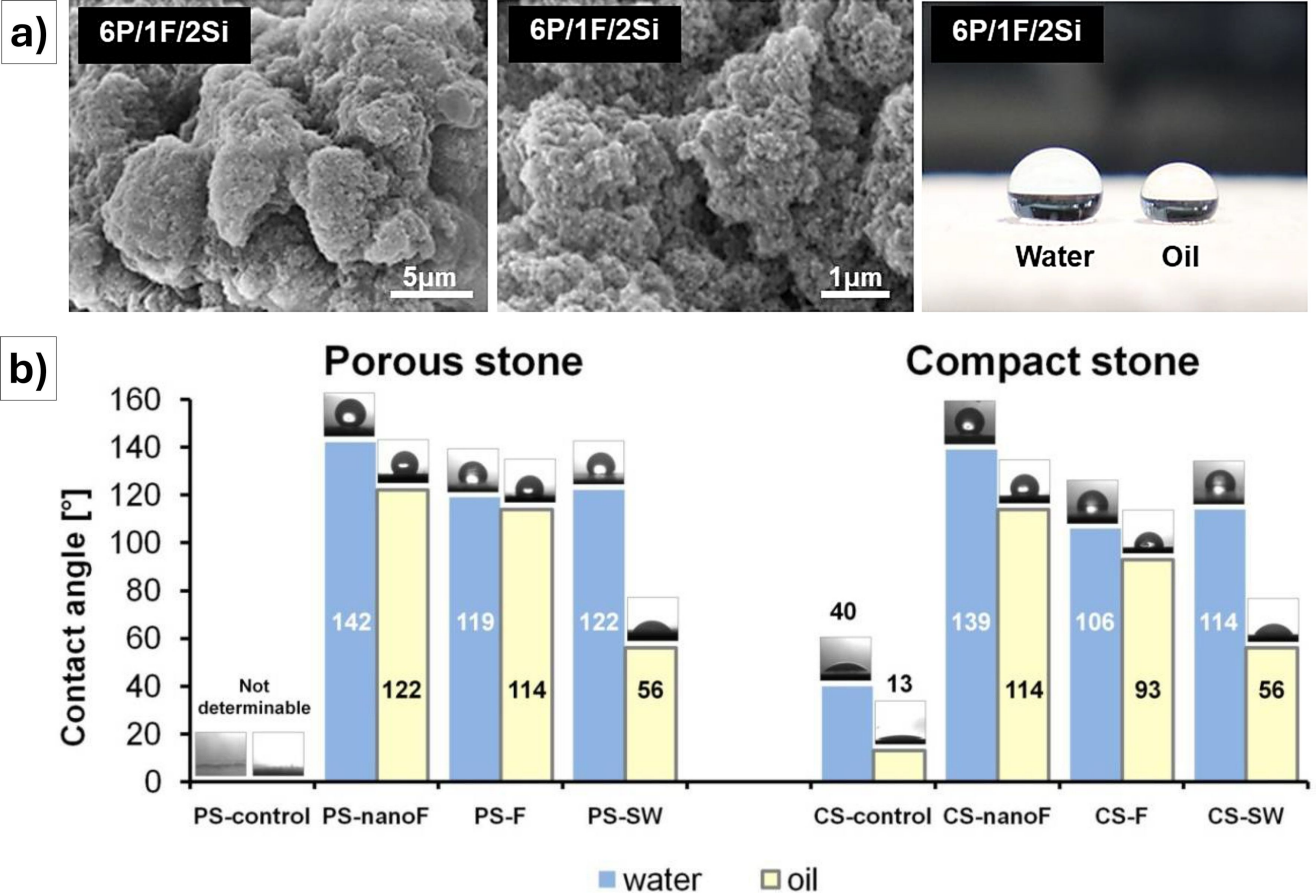
(a) High-magnification SEM images of the 6P/1F/2Si coating (left, center) produced in [115]. A photograph showing a water and oil drop on the 6P/1F/2Si surface is included (right). Figure 14a was adapted from [115] (© 2023, P.N. Manoudis et al., published by MDPI, distributed under the terms of the Creative Commons Attribution 4.0 International License, https://creativecommons.org/licenses/by/4.0). (b) Contact angle values; the water and oil droplets on the stone surfaces are also shown. Figure 14b was adapted from [116] (© 2021, M. Lettieri et al., published by MDPI, distributed under the terms of the Creative Commons Attribution 4.0 International License, https://creativecommons.org/licenses/by/4.0).

However, not much attention has been dedicated to oleophobic solutions in the field of heritage objects protection. Recent studies have advanced the development of superoleophobic coatings mostly for stone protection in cultural heritage applications. Manoudis et al. [115] and Lettieri et al. [116] followed similar approaches by combining low-surface-energy materials with nanoscale surface structuring. The former achieved true superoleophobicity (oil contact angle > 150°) on marble surfaces by blending a commercial silane product (Protectosil SC) with a fluoropolymer (Ruco-Guard) and SiO_2_ nanoparticles (7 nm), demonstrating that only the synergistic combination of surface roughness and chemical modification can produce superamphiphobic behavior (Figure 14a). In contrast, the latter applied a fluorine-based nanoparticle-filled coating (nanoF, containing SiO_2_ nanoparticles of 40–50 nm) on porous Lecce stone and dense Trani stone, achieving significant oleophobicity (oil contact angles > 100°) but not reaching the superoleophobic threshold. Nonetheless, nanoF outperformed commercial fluorinated and siloxane coatings in terms of oil repellency, water vapor permeability, anti-graffiti behavior, and resistance to simulated bird excreta (Figure 14b). Collectively, these studies underscore the potential of fluoropolymer–nanoparticle composites for multifunctional, durable protection of heritage stone, with superoleophobicity attainable through precise tuning of both material chemistry and micro/nanostructure.

Another great potential for reaching superliquiphobic/-philic systems, including also combinations of these features, is represented by the exploitation of Janus particles. Owing to their dual-sided nature, each side can exhibit distinct surface properties, enabling tailored interactions with different liquid phases [117–119]. Although Janus particles have found extensive applications in various scientific and technological domains [120–122], their potential in the field of cultural heritage protection remains largely untapped and highly promising.

## Summary and Concluding Remarks

In this review article, recent advances in the field of cultural heritage protection are reported. For decades, conservation science has relied on familiar polymer families (i.e., acrylics, polysiloxanes, fluoropolymers, polyurethanes, epoxies, and, more recently, bio-based resins such as ethyl cellulose and shellac). Each polymer family contributes distinct strengths to the preservation of mineral, metallic, wooden, and textile substrates. Acrylics are of importance because of their optical neutrality and long tradition in metal protection, while polysiloxanes stand out as some of the least intrusive, most compatible water-repellents for porous stone. Fluoropolymers still define the benchmark for repellency and weathering stability, but their environmental persistence increasingly collides with conservation ethics. Polyurethanes and epoxies, practical and robust in application, fall short when reversibility is considered. Bio-based polymers, by contrast, are emerging as some of the most promising candidates for a new generation of coatings – transparent, renewable, safe, and even self-healing, though their oleophobicity and long-term outdoor resilience remain to be fully proven. In Table 8, a comparative summary of widely used functional materials within this review is presented, associated to each ICOM criterion, with sustainability as additional indicator.

**Table 8:** Summary of the most commonly used polymers (and related characteristic functional materials) in the analyzed literature, associated to each of the ICOM criteria (optical transparency, reversibility, stability, safety), introducing sustainability as additional indicator. Superhydrophobicity/-oleophobicity and heritage applicability of the same functional materials are also reported.

Functional material (family)	C–F (fluoroalkyl/PFPE blocks)

typical host polymer(s) in papers	TEOS–FAS sols; PFDMS; PFDTES; PFPE-grafted oligoamides
optical transparency	yes on light stones/ceramics; non-whitening achieved with controlled nano-dispersion
reversibility	partial and unclear: good permanence; explicit, gentle removability rarely demonstrated
chemical/UV stability	high heat/UV/acid–base tolerance; strong weathering and corrosion barriers
safety (operator/substrate)	good in practice; authors note potential environmental/health concerns of F chemistries
sustainability	contentious: PFAS persistence concerns; some studies emphasize benign solvents
superhydrophobicity/-oleophobicity (water/oil)	water contact angles of 150°–175° and often low roll-off; occasionally near-superoleophobic
superhydrophobicity in heritage (optics, breathability, and removal)	color neutral on bright substrates; whitening risk mitigated by probe-sonicated SiO_2_; small vapor-permeability drop acceptable on marble; removability seldom validated long-term.

Functional material (family)	Si–O–Si (alkyl-siloxane/PDMS)

typical host polymer(s) in papers	DTMS/PDMS; TEOS-PDMS; water-borne siloxanes
optical transparency	generally good; ΔE* kept low with careful filler loadings
reversibility	partial: sol–gel networks can be difficult to remove; some “green sol–gel” routes aim at milder systems
chemical/UV stability	base polymer stable; UV can degrade alkyl-silane alone; nano-additives help to improve durability
safety (operator/substrate)	favorable; water-borne/low-toxicity routes available
sustainability	better than fluorinated analogues, especially water-borne
superhydrophobicity/-oleophobicity (water/oil)	superhydrophobicity possible via hierarchical roughness from SiO_2_/TiO_2_/Ca(OH)_2_; oleophobicity modest
superhydrophobicity in heritage (optics, breathability, removal)	risk of whitening from agglomerates; breathability often retained; UV stability improved by nano-UV shielding; removal not routinely proven at scale.

Functional material (family)	Methacrylate/acrylic esters

typical host polymer(s) in papers	paraloid-type and fluorinated methacrylates
optical transparency	good when thin; prone to yellowing/optical shifts on aging without stabilizers
reversibility	variable; redissolution by solvent is possible early, harder after aging
chemical/UV stability	non-fluorinated acrylics can photo-degrade; semi-fluorinated side chains can improve
safety (operator/substrate)	generally acceptable; depends on solvent package
sustainability	partial; fluorinated variants raise persistence questions
superhydrophobicity/-oleophobicity (water/oil)	superhydrophobicity feasible with fluorinated side-chains; non-F acrylics typically just hydrophobic
superhydrophobicity in heritage (optics, breathability, removal)	long-term UV/color stability is the key limitation; oleophobicity hinges on fluorination; whitening can occur on dark metals.

Functional material (family)	Urethane (–NH–CO–O–)

typical host polymer(s) in papers	PU with long-chain silanes (e.g., HDTMS)
optical transparency	good; thin films are equivalently efficient
reversibility	partial; crosslinked networks are hard to reverse
chemical/UV stability	good mechanical/chemical durability; UV resistance depends on formulation
safety (operator/substrate)	acceptable with proper solvents
sustainability	better than fluorinated compounds
superhydrophobicity/-oleophobicity (water/oil)	superhydrophobicity achievable (PU+SiO_2_/HDTMS) with good self-cleaning
superhydrophobicity in heritage (optics, breathability, removal)	often hydrophobic rather than oleophobic; optical neutrality generally good; removal strategies not standardized.

Functional material (family)	Epoxy (–CH–(OH)–CH_2_–O–)

typical host polymer(s) in papers	epoxy–silane–SiO_2_ hybrids
optical transparency	good at low filler loadings
reversibility	limited; crosslinking limits reversibility
chemical/UV stability	high mechanical/chemical durability; UV resistance requires stabilizers
safety (operator/substrate)	acceptable with care
sustainability	better than fluorinated compounds
superhydrophobicity/-oleophobicity (water/oil)	superhydrophobicity achievable when combined with nano-roughness; oleophobicity limited
superhydrophobicity in heritage (optics, breathability, removal)	can embrittle/yellow without stabilizers; careful formulation needed to avoid gloss/microtexture change.

Functional material (family)	Polysaccharide ether

typical host polymer(s) in papers	ethyl cellulose (EC)
optical transparency	good; films might appear uneven
reversibility	potentially better (weaker solvent-borne interactions)
chemical/UV stability	needs hydrophobe/roughness to resist water or UV
safety (operator/substrate)	good
sustainability	high
superhydrophobicity/-oleophobicity (water/oil)	superhydrophobicity achievable with fatty acids/roughness; mostly hydrophobic
superhydrophobicity in heritage (optics, breathability, removal)	environmentally attractive; durability lower than silicones/fluoropolymers; may need renewability plan.

Functional material (family)	Natural polyester/resin

typical host polymer(s) in papers	shellac (with stearic acid)
optical transparency	good; warm tone possible
reversibility	better early (alcohol-soluble), harder after aging
chemical/UV stability	UV/heat sensitivity unless protected
safety (operator/substrate)	good
sustainability	high
superhydrophobicity/-oleophobicity (water/oil)	superhydrophobicity achievable only with added roughness; oleophobicity weak
superhydrophobicity in heritage (optics, breathability, removal)	excellent sustainability profile; durability and maintenance cycles must be planned; avoid gloss shifts on dark substrates.

In the past decade, the field has clearly pivoted from coatings that simply stabilize or consolidate, to systems that actively defend. The rise of superhydrophobic and superoleophobic surfaces signals this shift. By enabling extreme water repellency, these coatings do not merely slow deterioration, they also block many of the agents of decay at their entry point. In Table 8, the superhydrophobic potentials of each representative functional material, are summarized. They limit salt migration, freeze–thaw cracking, microbial colonization, and atmospheric soiling, while reducing the need for frequent, often invasive cleaning. The range of functionalities associated with these surfaces (namely, self-cleaning, anti-icing, anti-graffiti, and anti-corrosion) opens a new horizon for preventive conservation, one where coatings are designed as active shields, rather than passive barriers.

The limitations that have long defined the field still remain, and in some ways they are amplified by the pursuit of superhydrophobicity. Reversibility, a cornerstone of conservation ethics, is rarely addressed and seldom demonstrated. Even coatings that appear optically neutral on pale stones or wood may induce perceptible shifts on darker substrates or under glossy finishes. And perhaps most importantly, sustainability remains unresolved. Fluorinated chemicals continue to deliver unrivalled performance, but they bring with them issues of persistence, toxicity, and regulatory constraint. Non-fluorinated alternatives (such as alkylsiloxanes, PDMS-based sol–gels, or bio-derived composites) are narrowing the performance gap through hierarchical roughness and better dispersion control, but they still face challenges of UV stability, oleophobicity, and large-scale, long-term validation.

Future advances will require an integration of these principles into coating design to develop coatings that are transparent and non-intrusive, reversible or removable with minimal impact on the original material, chemically and mechanically stable under harsh outdoor conditions, nontoxic to humans and environmentally benign, and demonstrably sustainable across the entire life cycle, from synthesis to disposal. Some needed research already exists. Fluorine-free alkylsilane–oxide textures on concrete show scalability and durability without sacrificing appearance. CaCO_3_- or Ca(OH)_2_-compatible siloxanes for calcareous stones marry performance with chemical affinity. EC/shellac and nanolignin composites illustrate how renewable polymers can achieve hydrophobic and even superhydrophobic protection while aligning with ecological responsibility. But, so far, no single system brings together all these qualities.

Ultimately, the challenge for conservation science is no longer whether we can reach extreme repellency, but whether we can do it responsibly. The future of protective coatings will depend on replacing persistent fluorinated moieties with PFAS-free chemicals, embedding “designed-to-disassemble” concepts that make reversibility real, and requiring standardized outdoor trials and life-cycle assessments as part of the research agenda. Only by balancing performance, superhydrophobic and other advanced coatings can move from the laboratory bench to the ethical and sustainable care of cultural heritage. In this sense, the coatings reviewed here do not just represent incremental improvements; they mark the contours of a new paradigm, one where durability, reversibility, safety, and sustainability are not competing goals, but integrated design parameters.

## Data Availability

Data sharing is not applicable as no new data was generated or analyzed in this study.
